# Supplementary material for: Effectiveness and Cost-Effectiveness of Case Management in Advanced Heart Failure Patients Attended in Primary Care: A Systematic Review and Meta-Analysis
Source: Int J Environ Res Public Health. 2022 Oct 24;19(21):13823. doi: 10.3390/ijerph192113823 (PMC9656967; doi:10.3390/ijerph192113823)
Supplement: Supplementary file 1 [file ijerph-19-13823-s001.zip › ijerph-1962545-supplementary.pdf]

Table S1. Excluded articles at full text level

Title	Year	Journal	Volume	Issue	Pages	Authors	Exclusion Reasons
Risk score-guided multidisciplinary team-based Care for Heart Failure Inpatients is associated with lower 30-day readmission and lower 30-day mortality	2019	Am Heart J	219		78-88	Horne,B.D et al	wrong population
Nurse-Led Collaborative Management Using Telemonitoring Improves Quality of Life and Prevention of Rehospitalization in Patients with Heart Failure	2019	Int Heart J	60	6	1293-1302	Mizukawa,M et al	wrong population
Implementation and Impact of Home-Based Cardiac Rehabilitation in a Veterans Affair Medical Center	2019	Mil Med	185	44352	e859-e863	Prasada,S et al	wrong population
Implementation of a Shared Medical Appointment as a Holistic Approach to CHF Management	2019	Holist Nurs Pract	33	6	354-359	Law,T et al	wrong population
The development and pilot study of a nurse-led HOMe-based HEart failure self-Management Programme (the HOM-HEMP) for patients with chronic heart failure, following Medical Research Council guidelines	2019	Eur J Cardiovasc Nurs	19	3	212-222	Jiang,Y et al	wrong population
Value of information analysis in telehealth for chronic heart failure management	2019	PLoS One	14	6	e0218083	Grustam,A.S et al	wrong population
Coordinated-Transitional Care for Veterans with Heart Failure and Chronic Lung Disease	2019	J Am Geriatr Soc	67	7	1502-1507	Reese,R.L et al	wrong population
Implementation of an intensified outpatient follow-up protocol improves outcomes in patients with ventricular assist devices	2019	Clin Res Cardiol	108	11	1197-1207	Hamed,S et al	wrong intervention
Effect of Patient-Centered Transitional Care Services on Clinical Outcomes in Patients Hospitalized for Heart Failure: The PACT-HF Randomized Clinical Trial	2019	JAMA	321	8	753-761	Van Spall,H.G.C et al	wrong population

Implications of Transitional Care Interventions on Hospital Readmissions in Patients With Destination Therapy Left Ventricular Assist Devices	2019	Res Theory Nurs Pract	33	1	81-96	Iseler,J.I et al	not found
Influence of Risk on Reduction of Readmission and Death by Disease Management Programs in Heart Failure	2019	J Card Fail	25	5	330-339	Huynh,Q.L et al	wrong population
Disease management interventions for heart failure	2019	Cochrane Database Syst Rev	1	1	CD002752	Takeda,A et al	systematic review
Effectiveness of a community care management program for multimorbid elderly patients with heart failure in the Veneto Region	2019	Aging Clin Exp Res	31	2	241-247	Tiozzo,S.N et al	wrong population
Effectiveness of the European Society of Cardiology/Heart Failure Association website 'heartfailurematters.org' and an e-health adjusted care pathway in patients with stable heart failure: results of the 'e-Vita HF' randomized controlled trial	2019	Eur J Heart Fail	21	2	238-246	Wagenaar,K.P et al	wrong population
Destination Therapy: Standardizing the Role of Palliative Medicine and Delineating the DT-LVAD Journey	2019	J Pain Symptom Manage	57	2	330-340	Woodburn,J.L et al	wrong intervention
A complex intervention of self-management for patients with COPD or CHF in primary care improved performance and satisfaction with regard to own selected activities; A longitudinal follow-up	2019	J Adv Nurs	75	1	175-186	Zakrisson,A.B et al	wrong population
Introducing nurse-led heart failure clinics in Swedish primary care settings	2019	Eur J Heart Fail	21	1	103-109	Liljeroos,M et al	wrong population
Evaluation of a nurse-led intervention program in heart failure: A randomized trial	2019	Med Clin (Barc)	152	11	431-437	Ortiz-Bautista,C et al	wrong population
The Effect of a Telephone-Based Self-management Program Led by Nurses on Self-care Behavior, Biological Index for Cardiac Function, and Depression in Ambulatory Heart Failure Patients	2018	Asian Nurs Res (Korean Soc Nurs Sci)	12	4	251-257	Moon,M.K et al	wrong population

Impact of telemedicine on the clinical outcomes and healthcare costs of patients with chronic heart failure and mid-range or preserved ejection fraction managed in a multidisciplinary chronic heart failure programme: A sub-analysis of the iCOR randomized trial	2020	J Telemed Telecare	24	1_2	65-72	Jimenez-Marrero,S et al	wrong population
Effects of a person-centred telephone-support in patients with chronic obstructive pulmonary disease and/or chronic heart failure - A randomized controlled trial	2018	PLoS One	13	8	e0203031	Fors,A et al	wrong population
Achieving IHI's Triple Aim by Utilizing Core Health Program With Community Health Workers in Rural Communities	2018	Fam Community Health	41	4	255-264	Fawcett,K.J.,Jr et al	wrong population
Effects of a multidisciplinary disease management programme with or without exercise training for heart failure patients: Secondary analysis of a randomized controlled trial	2018	Int J Nurs Stud	87		94-102	Liu,M.H et al	wrong population
Impact of the Implementation of Project Re-Engineered Discharge for Heart Failure patients at a Veterans Affairs Hospital at the Central Arkansas Veterans Healthcare System	2018	Hosp Pharm	53	4	266-271	Patel,P.H et al	wrong population
Heart Failure Management in Nursing Homes: A Scoping Literature Review	2018	Can J Cardiol	34	7	871-880	Heckman,G.A et al	systematic review
A review of integrated heart failure care	2018	Prim Health Care Res Dev	20		e57	MacInnes,J et al	systematic review
Opposite trends in hospitalization and mortality after implementation of a chronic care model-based regional program for the management of patients with heart failure in primary care	2018	BMC Health Serv Res	18	1	388	Ballo,P et al	wrong population
Effectiveness of a transition plan at discharge of patients hospitalized with heart failure: a before-and-after study	2018	ESC Heart Fail	5	4	657-667	Garnier,A et al	wrong population

Cost-Effectiveness of a Home Visit Program for Patients with Heart Failure in Brazil: Evidence from a Randomized Clinical Trial	2018	Value Health Reg Issues	17		81-87	Ruschel,K.B et al	wrong population
Effect of Grand-Aides Nurse Extenders on Readmissions and Emergency Department Visits in Medicare Patients With Heart Failure	2018	Am J Cardiol	121	11	1336-1342	Thomas,S.C et al	wrong population
Performance Improvement to Decrease Readmission Rates for Patients With a Left Ventricular Assist Device	2018	Prog Transplant	28	2	184-188	Iseler,J et al	wrong population
[Efficacy of a multidisciplinary care management program for patients admitted at hospital because of heart failure (ProMIC)]	2019	Aten Primaria	51	3	142-152	Domingo,C et al	wrong population
Effect of a Collaborative Care Intervention vs Usual Care on Health Status of Patients With Chronic Heart Failure: The CASA Randomized Clinical Trial	2018	JAMA Intern Med	178	4	511-519	Bekelman,D.B et al	wrong population
The first multicenter, randomized, controlled trial of home telemonitoring for Japanese patients with heart failure: home telemonitoring study for patients with heart failure (HOMES-HF)	2018	Heart Vessels	33	8	866-876	Kotooka,N et al	wrong population
Videoconferencing for Management of Heart Failure: An Integrative Review	2018	J Gerontol Nurs	44	4	45-52	Bauce,K et al	systematic review
Pilot testing of the effectiveness of nurse-guided, patient-centered heart failure education for older adults	2018	Geriatr Nurs	39	4	376-381	Mathew,S et al	wrong population
The effect of nurse-led education on hospitalisation, readmission, quality of life and cost in adults with heart failure. A systematic review	2018	Patient Educ Couns	101	3	363-374	Rice,H et al	systematic review
Home-based telerehabilitation in older patients with chronic obstructive pulmonary disease and heart failure: a randomised controlled trial	2018	Age Ageing	47	1	82-88	Bernocchi,P et al	wrong population
Palliative Care in Heart Failure: Rationale, Evidence, and Future Priorities	2017	J Am Coll Cardiol	70	15	1919-1930	Kavalieratos,D et al	systematic review

Telemanagement of Heart Failure Patients Across the Post-Acute Care Continuum	2018	Telemed J E Health	24	5	360-366	Dadosky,A et al	wrong population
Processes and Outcomes of Congestive Heart Failure Care by Different Types of Primary Care Models	2018	J Card Fail	24	1	9-18	Kuo,Y.F et al	wrong population
An evaluation of involving family caregivers in the self-care of heart failure patients on hospital readmission: Randomised controlled trial (the FAMILY study)	2017	Int J Nurs Stud	75		101-111	Deek,H et al	wrong population
Implementation of a Patient Navigator Program to Reduce 30-day Heart Failure Readmission Rate	2017	Prog Cardiovasc Dis	60	2	259-266	Di Palo,K.E et al	wrong population
Effect of Early Follow-Up After Hospital Discharge on Outcomes in Patients With Heart Failure or Chronic Obstructive Pulmonary Disease: A Systematic Review	2017	Ont Health Technol Assess Ser	17	8	1-37	Song, J et al	wrong population
Impact of a Multidisciplinary Heart Failure Postdischarge Management Clinic on Medication Adherence	2017	Clin Ther	39	6	1200-1209	Lu, L et al	wrong population
Standard vs. intensified management of heart failure to reduce healthcare costs: results of a multicentre, randomized controlled trial	2017	Eur Heart J	38	30	2340-2348	Scuffham,P.A et al	wrong population
Disease management in the treatment of patients with chronic heart failure who have universal access to health care: a randomized controlled trial	2017	BMC Med	15	1	90	Kalter-Leibovici,O et al	wrong population
[The APULIA HF multicenter study: efficacy of a management protocol shared between hospital and territorial health services for acute decompensated heart failure patients]	2017	G Ital Cardiol (Rome)	18	2	150-158	Iacoviello,M et al	wrong population
Transitional care interventions: Relevance for nursing in the community	2017	Public Health Nurs	34	5	454-460	Coffey,A et al	wrong population
The effectiveness of telemedicine in the management of chronic heart disease - a systematic review	2017	JRSM Open	8	3	2054270416 681747	Kruse,C.S et al	systematic review

Comparative effectiveness of transitional care services in patients discharged from the hospital with heart failure: a systematic review and network meta-analysis	2017	Eur J Heart Fail	19	11	1427-1443	Van Spall,H.G.C et al	systematic review
Cost-effectiveness analysis for a tele-based health coaching program for chronic disease in primary care	2017	BMC Health Serv Res	17	1	138	Oksman,E et al	wrong population
Remote Monitoring of Patients With Heart Failure: An Overview of Systematic Reviews	2017	J Med Internet Res	19	1	e18	Bashi,N et al	systematic review
[Development and Effects of a Heart Health Diary for Self-Care Enhancement of Patients with Heart Failure]	2016	J Korean Acad Nurs	46	6	881-893	Shim,J.L et al	wrong intervention
Improving quality of life and decreasing readmissions in heart failure patients in a multidisciplinary transition-to-care clinic	2017	Heart Lung	46	2	79-84	Whitaker-Brown,C.D et al	wrong population
A Retrospective Comparison of Home Telehealth and Nursing Care With or Without Rehabilitation Therapy on Rehospitalization Rates of Individuals With Heart Failure	2017	J Cardiopulm Rehabil Prev	37	3	207-213	Martin,S et al	wrong population
What is the impact of systems of care for heart failure on patients diagnosed with heart failure: a systematic review	2016	BMC Cardiovasc Disord	16	1	195	Driscoll,A et al	systematic review
The Intensity of Primary Care for Heart Failure Patients: A Determinant of Readmissions? The CarPaths Study: A French Region-Wide Analysis	2016	PLoS One	11	10	e0163268	Duflos,C.M et al	wrong population
[Reduction of rehospitalisation on elderly heart failure patients: A retrospective cohort VISage network]	2016	Ann Cardiol Angeiol (Paris)	65	5	293-298	Labranche,D et al	wrong population
Feasibility study of a nurse-led heart failure education program	2016	Contemp Nurse	52	4	499-510	Baptiste,D.L et al	wrong population
Reducing Readmissions among Heart Failure Patients Discharged to Home Health Care:	2017	Health Serv Res	52	4	1445-1472	Murtaugh,C.M et al	wrong population

Effectiveness of Early and Intensive Nursing Services and Early Physician Follow-Up							
The E-Coach technology-assisted care transition system: a pragmatic randomized trial	2016	Transl Behav Med	6	3	428-437	Ritchie,C.S et al	wrong population
A High-Touch Model of Community-Based Specialist Palliative Care: Latent Class Analysis Identifies Distinct Patient Subgroups	2016	J Pain Symptom Manage	52	2	178-186	Dhingra,L et al	wrong study design
Does case management for patients with heart failure based in the community reduce unplanned hospital admissions? A systematic review and meta-analysis	2016	BMJ Open	6	5	e010933	Huntley,A.L et al	systematic review
Health-Related Quality of Life in Heart Failure Patients With Varying Levels of Health Literacy Receiving Telemedicine and Standardized Education	2016	Home Healthc Now	34	5	267-272	Yehle,K.S et al	wrong population
Evaluation of the efficacy of a nurse practitioner-led home-based congestive heart failure clinical pathway	2016	Home Health Care Serv	35	1	39-51	Moore,J.A	wrong population
Effects of a transitional palliative care model on patients with end-stage heart failure: a randomised controlled trial	2016	Heart	102	14	1100-1108	Wong,F.K et al	wrong population
Evaluation of a Community Health Service Center-Based Intervention Program for Managing Chronic Heart Failure	2016	Balkan Med J	33	1	45-51	Gu,M et al	wrong population
Decreasing Congestive Heart Failure Readmission Rates Within 30 Days at the Tampa VA	2016	Nurs Adm Q	40	2	146-152	Messina,W	wrong population
How effective is an in-hospital heart failure self-care program in a Japanese setting? Lessons from a randomized controlled pilot study	2016	Patient Prefer Adherence	10		171-181	Kato,N.P et al	wrong population
Nurse Practitioner Care Model: Meeting the Health Care Challenges With a Collaborative Team	2015	Nurs Econ	33	6	297-304	Kutzleb,J et al	not found
A systematic review of transitional-care strategies to reduce rehospitalization in patients with heart failure	2016	Heart Lung	45	2	100-113	Albert,N.M.	systematic review

Protocol-Driven Allied Health Post-Discharge Transition Clinic to Reduce Hospital Readmissions in Heart Failure	2015	J Am Heart Assoc	4	12		Donaho,E.K et al	wrong population
Impact of APN Home Visits in Reducing Healthcare Costs and Improving Function in Homebound Heart Failure	2015	Home Healthc Now	33	10	532-537	Echeverry,L.M et al	wrong population
Effectiveness of an interactive platform, and the ESC/HFA heartfailurematters.org website in patients with heart failure: design of the multicentre randomized e-Vita heart failure trial	2015	Eur J Heart Fail	17	12	1310-1316	Wagenaar,K.P et al	wrong population
Combining training in knowledge translation with quality improvement reduced 30-day heart failure readmissions in a community hospital: a case study	2016	J Eval Clin Pract	22	2	171-179	Wyer,P et al	wrong population
Interactive telemedicine: effects on professional practice and health care outcomes	2015	Cochrane Database Syst Rev		9	CD002098	Flodgren,G et al	systematic review
Evaluation of telehealth service for patients with congestive heart failure in the north of Israel	2016	Eur J Cardiovasc Nurs	15	3	e78-e84	Eilat-Tsanani,S et al	wrong population
Cost-effectiveness of home versus clinic-based management of chronic heart failure: Extended follow-up of a pragmatic, multicentre randomized trial cohort - The WHICH? study (Which Heart Failure Intervention Is Most Cost-Effective & Consumer Friendly in Reducing Hospital Care)	2015	Int J Cardiol	201		368-375	Maru,S et al	wrong population
Effect of Nurse-Implemented Transitional Care for Chinese Individuals with Chronic Heart Failure in Hong Kong: A Randomized Controlled Trial	2015	J Am Geriatr Soc	63	8	1583-1593	Yu,D.S et al	wrong population
Impact of a Multidisciplinary Heart Failure Post-hospitalization Program on Heart Failure Readmission Rates	2015	Ann Pharmacother	49	11	1189-1196	Jackevicius,C.A et al	wrong population
The clinical effectiveness and cost-effectiveness of clinical nurse specialist-led hospital to home transitional care: a systematic review	2015	J Eval Clin Pract	21	5	763-781	Bryant-Lukosius,D et al	systematic review

Outcome of Patients Discharged From a Heart Failure Disease Management Program following Their Clinical and Echocardiographic Recovery	2015	Cardiology	131	3	197-202	Proctor,P et al	wrong population
Heart failure remote monitoring: evidence from the retrospective evaluation of a real-world remote monitoring program	2015	J Med Internet Res	17	4	e101	Agboola,S et al	wrong population
Seamless Transitions: Achieving Patient Safety Through Communication and Collaboration	2018	J Patient Saf	14	1	e3-e5	Radhakrishnan,K et al	wrong population
Nurse-Led Multidisciplinary Heart Failure Group Clinic Appointments: Methods, Materials, and Outcomes Used in the Clinical Trial	2015	J Cardiovasc Nurs	30	4	S25-S34	Smith,C.E et al	wrong population
Telecare for diabetes, CHF or COPD: effect on quality of life, hospital use and costs. A randomised controlled trial and qualitative evaluation	2015	PLoS One	10	3	e0116188	Kenealy,T.W et al	wrong population
If home telemonitoring reduces mortality in heart failure, is this just due to better guideline-based treatment?	2015	J Telemed Telecare	21	6	331-339	Dierckx,R et al	wrong population
Care in the Home for the Management of Chronic Heart Failure: Systematic Review and Cost-Effectiveness Analysis	2015	J Cardiovasc Nurs	30	4	S44-S51	Fergenbaum,J et al	systematic review
Home telehealth and hospital readmissions: a retrospective OASIS-C data analysis	2015	Home Healthc Now	33	1	20-26	Thomason,T.R et al	wrong population
A heart failure initiative to reduce the length of stay and readmission rates	2014	Prof Case Manag	19	6	276-284	White,S.M et al	wrong population
Resource use and cost implications of implementing a heart failure program for patients with systolic heart failure in Swedish primary health care	2014	Int J Cardiol	176	3	731-738	Agvall,B et al	wrong population
A nurse-based strategy reduces heart failure morbidity in patients admitted for acute decompensated heart failure in Brazil: the HELEN-II clinical trial	2014	Eur J Heart Fail	16	9	1002-1008	de Souza,E.N et al	wrong population

[Early implementation of home care and 30 day readmissions in >65 years Veneto region patients discharged for heart failure and with disability]	2014	Assist Inferm Ric	33	2	67-73	Gennaro,N et al	not found
Transitional care interventions to prevent readmissions for persons with heart failure: a systematic review and meta-analysis	2014	Ann Intern Med	160	11	774-784	Feltner,C et al	systematic review
Efficacy of an integrated hospital-primary care program for heart failure: a population-based analysis of 56,742 patients	2014	Rev Esp Cardiol (Engl Ed)	67	4	283-293	Comin-Colet,J et al	wrong population
The effect of a randomized trial of home telemonitoring on medical costs, 30-day readmissions, mortality, and health-related quality of life in a cohort of community-dwelling heart failure patients	2014	J Card Fail	20	7	513-521	Blum,K et al	wrong population
The comparison of the effects of education provided by nurses on the quality of life in patients with congestive heart failure (CHF) in usual and home-visit cares in Iran	2014	Glob J Health Sci	6	3	256-260	Mehralian,H et al	wrong population
Integrated telehealth care for chronic illness and depression in geriatric home care patients: the Integrated Telehealth Education and Activation of Mood (I-TEAM) study	2014	J Am Geriatr Soc	62	5	889-895	Gellis,Z.D et al	wrong population
Cost-effectiveness of a nurse facilitated, cognitive behavioural self-management programme compared with usual care using a CBT manual alone for patients with heart failure: secondary analysis of data from the SEMAPHFOR trial	2014	Int J Nurs Stud	51	9	1214-1220	Mejia,A et al	wrong population
The effect of multidisciplinary heart failure clinic characteristics on 1-year postdischarge health care costs: a population-based study	2014	Med Care	52	3	272-279	Wijeysundera,H.C et al	wrong population

Nurse facilitated Self-management support for people with heart failure and their family carers (SEMAPHFOR): a randomised controlled trial	2014	Int J Nurs Stud	51	9	1207-1213	Cockayne,S et al	wrong population
Examining the effects of remote monitoring systems on activation, self-care, and quality of life in older patients with chronic heart failure	2015	J Cardiovasc Nurs	30	1	51-57	Evangelista,L.S et al	wrong population
Feasibility and acceptability of a collaborative care intervention to improve symptoms and quality of life in chronic heart failure: mixed methods pilot trial	2014	J Palliat Med	17	2	145-151	Bekelman,D.B et al	wrong population
Home-based telesurveillance program in chronic heart failure: effects on clinical status and implications for 1-year prognosis	2013	Telemed J E Health	19	8	605-612	Giordano,A et al	wrong population
The effect of telemonitoring at home on quality of life and self-care behaviors of patients with heart failure	2013	Home Healthc Nurse	31	7	368-377	Hoban,M.B et al	wrong population
Impact of telemonitoring home care patients with heart failure or chronic lung disease from primary care on healthcare resource use (the TELBIL study randomised controlled trial)	2013	BMC Health Serv Res	13		118	Martin-Lesende,I et al	wrong population
Transitional care programs improve outcomes for heart failure patients: an integrative review	2014	J Cardiovasc Nurs	29	2	140-154	Stamp,K.D et al	systematic review
Cost-utility analysis of nt-probnp-guided multidisciplinary care in chronic heart failure	2013	Int J Technol Assess Health Care	29	1	3-11	Moertl,D et al	wrong population
A population-based study to evaluate the effectiveness of multidisciplinary heart failure clinics and identify important service components	2013	Circ Heart Fail	6	1	68-75	Wijeysundera,H.C et al	wrong population
The benefits of using a heart failure management programme in Swedish primary healthcare	2013	Eur J Heart Fail	15	2	228-236	Agvall,B et al	wrong population
Telephone support to rural and remote patients with heart failure: the Chronic Heart Failure Assessment by Telephone (CHAT) study	2013	Cardiovasc Ther	31	4	230-237	Krum,H et al	wrong population
Impact of home versus clinic-based management of chronic heart failure: the WHICH? (Which Heart	2012	J Am Coll Cardiol	60	14	1239-1248	Stewart,S et al	wrong population

Failure Intervention Is Most Cost-Effective & Consumer Friendly in Reducing Hospital Care) multicenter, randomized trial							
A critical review on telemonitoring in heart failure	2012	Acta Cardiol	67	4	439-444	Gurne,O et al	systematic review
Clinical service organisation for heart failure	2012	Cochrane Database Syst Rev		9	CD002752	Takeda,A et al	systematic review
Role of a multidisciplinary program in improving outcomes in cognitively impaired heart failure older patients	2012	Monaldi Arch Chest Dis	78	1	20-28	Del,Sindaco D et al	wrong intervention
A systematic review of economic evaluations of cardiac rehabilitation	2012	BMC Health Serv Res	12		243	Wong,W.P et al	systematic review
Effect of a community-based nursing intervention on mortality in chronically ill older adults: a randomized controlled trial	2012	PLoS Med	9	7	e1001265	Coburn,K.D et al	wrong population
The impact of remote patient monitoring (telehealth) upon Medicare beneficiaries with heart failure	2012	Telemed J E Health	18	2	101-108	Pekmezaris,R et al	wrong population
Evaluation of a nurse practitioner disease management model for chronic heart failure: a multi-site implementation study	2012	Congest Heart Fail	18	1	64-71	Lowery,J et al	wrong population
[CorBene: a new model for collaborative care of patients with congestive heart failure]	2012	Herz	37	1	56-58	Gysan,D.B et al	systematic review
[Home-based telemonitoring of simple vital signs to reduce hospitalization in heart failure patients: real-world data from a community-based hospital]	2011	G Ital Cardiol (Rome)	12	12	829-836	Palmieri,V et al	not found
Effectiveness of an educational self-management program for outpatients with chronic heart failure	2011	Jpn J Nurs Sci	8	2	140-152	Otsu,H et al	wrong population
Effectiveness of a self-care program in improving symptom distress and quality of life in congestive heart failure patients: a preliminary study	2011	J Nurs Res	19	4	257-266	Wang,S.P et al	wrong population
Home-based telemanagement in chronic heart failure: an 8-year single-site experience	2011	J Telemed Telecare	17	7	382-386	Giordano,A et al	wrong population

Clinical outcome of patients with chronic heart failure followed in a specialized heart failure center	2011	Isr Med Assoc J	13	8	468-473	Gotsman,I et al	wrong population
Automated home telephone self-monitoring reduces hospitalization in patients with advanced heart failure	2011	J Telemed Telecare	17	6	298-302	Kurtz,B et al	wrong population
Effectiveness and cost of a transitional care program for heart failure: a prospective study with concurrent controls	2011	Arch Intern Med	171	14	1238-1243	Stauffer,B.D et al	wrong population
Rationale and design of the Japanese heart failure outpatients disease management and cardiac evaluation (J-HOMECARE)	2011	J Cardiol	58	2	165-172	Tsuchihashi-Makaya,M et al	wrong publication type
Home monitoring cuts cardiac readmissions	2011	Hosp Case Manag	19	5	76-77	No authors listed	not found
Discharge to a skilled nursing facility and subsequent clinical outcomes among older patients hospitalized for heart failure	2011	Circ Heart Fail	4	3	293-300	Allen,L.A et al	wrong population
Swiss Interdisciplinary Management Programme for Heart Failure (SWIM-HF): a randomised controlled trial study of an outpatient inter-professional management programme for heart failure patients in Switzerland	2011	Swiss Med Wkly	141		w13171	Leventhal,M.E et al	wrong population
The impact of proactive chronic care management on hospital admissions in a German senior population	2011	Popul Health Manag	14		S29-S33	Hamar,B et al	wrong population
The effect of telehomecare on heart failure self care	2010	AMIA Annu Symp Proc	2010		71-75	Bowles,K.H et al	wrong population
Education and telephone monitoring by nurses of patients with heart failure: randomized clinical trial	2011	Arq Bras Cardiol	96	3	233-239	Domingues,F.B et al	wrong population
Health-related quality of life in a multicenter randomized controlled comparison of telephonic disease management and automated home monitoring in patients recently hospitalized with heart failure: SPAN-CHF II trial	2011	J Card Fail	17	2	151-157	Konstam,V et al	wrong population

Economic impact of remote patient monitoring: an integrated economic model derived from a meta-analysis of randomized controlled trials in heart failure	2011	Eur J Heart Fail	13	4	450-459	Klersy,C et al	systematic review
Telehealth in adult patients with congestive heart failure in long term home health care: a systematic review	2011	JBI Libr Syst Rev	9	30	1271-1296	Cherofsky,N et al	systematic review
Evaluation of the transitional care model in chronic heart failure	2010	Br J Nurs	19	22	1402-1407	Williams,G et al	wrong population
Cost-effectiveness of specialized multidisciplinary heart failure clinics in Ontario, Canada	2010	Value Health	13	8	915-921	Wijeysundera,H.C et al	wrong population
A randomized trial of telemonitoring heart failure patients	2010	J Healthc Manag	55	5	312-322	Tompkins,C et al	wrong population
The impact of a nurse-led care programme on events and physical and psychosocial parameters in patients with heart failure with preserved ejection fraction: a randomized clinical trial in primary care in Russia	2010	Eur J Gen Pract	16	4	205-214	Andryukhin,A et al	wrong population
Improved quality of life in Norwegian heart failure patients after follow-up in outpatient heart failure clinics: results from the Norwegian Heart Failure Registry	2010	Eur J Heart Fail	12	11	1247-1252	Hole,T et al	wrong population
Long-term implications of a single home-based educational intervention in patients with heart failure	2010	Heart Lung	39	6	S14-S22	Aguado,O et al	wrong population
Can a heart failure-specific cardiac rehabilitation program decrease hospitalizations and improve outcomes in high-risk patients?	2010	Eur J Cardiovasc Prev Rehabil	17	4	393-402	Davidson,P.M et al	wrong population
Nurse-led self-management group programme for patients with congestive heart failure: randomized controlled trial	2010	J Adv Nurs	66	7	1487-1499	Smeulders,E.S et al	wrong population
Case management for patients with chronic systolic heart failure in primary care: the HICMan exploratory randomised controlled trial	2010	Trials	11		56	Peters-Klimm,F et al	wrong population

Randomized trials of nursing interventions for secondary prevention in patients with coronary artery disease and heart failure: systematic review	2010	J Cardiovasc Nurs	25	3	207-220	Allen,J.K et al	systematic review
[Comparison between telephone and outpatient nursing management in patients with chronic heart failure in a large territorial area in Piedmont, Italy]	2010	G Ital Cardiol (Rome)	11	1	35-42	Mainardi,L et al	not found
A multicenter randomized controlled evaluation of automated home monitoring and telephonic disease management in patients recently hospitalized for congestive heart failure: the SPAN-CHF II trial	2010	J Card Fail	16	4	285-292	Weintraub,A et al	wrong population
Assessment of the clinical outcomes and cost-effectiveness of the management of systolic heart failure in Chinese patients using a home-based intervention	2010	J Int Med Res	38	1	242-252	Chen,Y.H et al	wrong population
Nurse-led interventions in heart failure care: patient and nurse perspectives	2010	Eur J Cardiovasc Nurs	9	4	226-232	Hoekstra,T et al	wrong population
Evidence-based nursing: the role of the advanced practice registered nurse in the management of heart failure patients in the outpatient setting	2010	Dimens Crit Care Nurs	29	2	57-62	Case,R et al	systematic review
Efficacy of multidisciplinary outpatient management (MOM) program in long term heart failure care	2010	South med J	103	2	131-137	Jain,R et al	wrong population
Primary care-based multifaceted, interdisciplinary medical educational intervention for patients with systolic heart failure: lessons learned from a cluster randomised controlled trial	2009	Trials	10		68	Peters-Klimm,F et al	wrong population
[Integrated care for patients with heart failure in Switzerland: a cost analysis]	2009	Praxis (Bern 1994)	98	15	809-815	Eichler,K et al	wrong population
Improving outcomes for older adults with heart failure: a randomized trial using a theory-guided nursing intervention	2010	J Nurs Care Qual	25	1	56-64	Duffy,J.R et al	wrong population
Outcomes of a home telehealth intervention for patients with heart failure	2009	J Telemed Telecare	15	1	46-50	Wakefield,B.J et al	wrong population

Community-based care for the specialized management of heart failure: an evidence-based analysis	2009	Ont Health Technol Assess Ser	9	17	1-42	Medical Advisory Secretariat	systematic review
Cost-effectiveness of nurse-led disease management for heart failure in an ethnically diverse urban community	2008	Ann Intern Med	149	8	540-548	Hebert,P.L et al	wrong population
[Value of basic and intensive management of patients with heart failure; results of a randomised controlled clinical trial]	2008	Ned Tijdschr Geneeskd	152	37	2016-2021	Jaarsma,T et al	not found
Tele-guidance of chronic heart failure patients enhances knowledge about the disease. A multi-centre, randomised controlled study	2008	Eur J Heart Fail	10	11	1136-1142	Balk,A.H et al	wrong population
Impact of telehealth on clinical outcomes in patients with heart failure	2008	Clin Nurs Res	17	3	182-199	Dansky,K.H et al	wrong population
Multidisciplinary management of elderly patients with chronic heart failure: five year outcome measures in death and survivor groups	2009	Eur J Cardiovasc Nurs	8	1	34-39	Austin,J et al	wrong population
Cost-effectiveness of a disease management programme for secondary prevention of coronary heart disease and heart failure in primary care	2008	Heart	94	12	1601-1606	Turner,D.A et al	wrong population
Survival and hospitalization in a nurse-led domiciliary intervention for elderly heart failure patients	2008	J Cardiovasc Med (Hagerstown)	9	5	470-475	Rondinini,L et al	wrong population
Five-year follow-up findings from a randomized controlled trial of cardiac rehabilitation for heart failure	2008	Eur J Cardiovasc Prev Rehabil	15	2	162-167	Austin,J et al	wrong population
Cost-effective care a phone call away: a nurse-managed telephonic program for patients with chronic heart failure	2008	Nurs Econ	26	1	41-44	Slater,M.R et al	not found
Telemonitoring of heart failure patients and their caregivers: a pilot randomized controlled trial	2008	Prog Cardiovasc Nurs	23	1	18-26	Schwarz,K.A et al	wrong population

Lessons learned from a multidisciplinary heart failure clinic for older women: a randomised controlled trial	2008	Age Ageing	37	3	282-287	Azad,N et al	wrong population
Effect of moderate or intensive disease management program on outcome in patients with heart failure: Coordinating Study Evaluating Outcomes of Advising and Counseling in Heart Failure (COACH)	2008	Arch Intern Med	168	3	316-324	Jaarsma,T et al	wrong population
Multicenter randomised trial on home-based telemanagement to prevent hospital readmission of patients with chronic heart failure	2009	Int J Cardiol	131	2	192-199	Giordano,A et al	wrong population
Use of telehealth by older adults to manage heart failure	2008	Res Gerontol Nurs	1	1	25-32	Dansky,K.H et al	not found
A randomized controlled trial of a community nurse-supported hospital discharge programme in older patients with chronic heart failure	2008	J Clin Nurs	17	1	109-117	Kwok,T et al	wrong population
Impact of the implementation of telemanagement on a disease management program in an elderly heart failure cohort	2007	Prog Cardiovasc Nurs	22	4	196-200	Gambetta,M et al	wrong population
A systematic review of nurse-assisted case management to improve hospital discharge transition outcomes for the elderly	2007	Prof Case Manag	12	6	330-336	Chiu,W.K et al	systematic review
The use of supportive-educative and mutual goal-setting strategies to improve self-management for patients with heart failure	2007	Home Healthc Nurse	25	8	502-510	Kline,K.S et al	wrong population
Impact of a specialized outpatient heart failure follow-up program on hospitalization frequency and functional status of patients with advanced heart failure	2007	Rev Port Cardiol	26	4	335-343	Correia,J et al	wrong intervention
[Heart failure: the importance of a disease management program]	2007	G Ital Cardiol (Rome)	8	6	353-358	Fabbri,G et al	not found
A systematic review of the benefits of home telecare for frail elderly people and those with long-term conditions	2007	J Telemed Telecare	13	4	172-179	Barlow,J et al	systematic review

Efficacy of community-based multidisciplinary disease management of chronic heart failure	2007	Singapore Med J	48	6	528-531	Omar,A.R et al	wrong population
Lack of long-term benefits of a 6-month heart failure disease management program	2007	J Card Fail	13	4	287-293	Nguyen,V et al	wrong population
Improved cost-effectiveness for management of chronic heart failure by combined home-based intervention with clinical nursing specialists	2007	J Formos Med Assoc	106	4	313-319	Ho,Y.L et al	wrong population
Two-year outcome of a prospective, controlled study of a disease management programme for elderly patients with heart failure	2007	J Cardiovasc Med (Hagerstown)	8	5	324-329	Del,Sindaco D et al	wrong population
[New multidisciplinary heart failure care program (six-month preliminary observation)]	2006	Pol Merkur Lekarski	21	126	511-515	Wierzchowiecki, M et al	not found
Home-based palliative care study: site of death, and costs of medical care for patients with congestive heart failure, chronic obstructive pulmonary disease, and cancer	2005	J Soc Work End Life Palliat Care	1	3	37-56	Enguidanos,S.M et al	wrong intervention
The effect of frontloading visits on patient outcomes	2007	Home Healthc Nurse	25	2	103-109	Rogers,J et al	wrong population
Motivational interviewing to change quality of life for people with chronic heart failure: a randomised controlled trial	2008	Int J Nurs Stud	45	4	489-500	Brodie,D.A et al	wrong population
Applying research evidence to optimize telehomecare	2007	J Cardiovasc Nurs	22	1	5-15	Bowles,K.H et al	systematic review
Extending the horizon in chronic heart failure: effects of multidisciplinary, home-based intervention relative to usual care	2006	Circulation	114	23	2466-2473	Inglis,S.C. et al	wrong population
Advanced practice nurse strategies to improve outcomes and reduce cost in elders with heart failure	2006	Dis Manag	9	5	302-310	McCauley,K.M et al	systematic review
Telephone follow-up of self-care behaviour after a single session education of patients with heart failure in primary health care	2007	Eur J Cardiovasc Nurs	6	2	153-159	Holst,M et al	wrong population
Effects of nurse management on the quality of heart failure care in minority communities: a randomized trial	2006	Ann Intern Med	145	4	273-283	Sisk,J.E et al	wrong population

Telephone interventions by nursing students: improving outcomes for heart failure patients in the community	2006	J Community Health Nurs	23	3	137-146	Wheeler,E.C et al	wrong population
Exercise self-efficacy in older women with diastolic heart failure: results of a walking program and education intervention	2006	J Gerontol Nurs	32	7	31-39	Gary,R.	not found
Lack of improvement of clinical outcomes by a low-cost, hospital-based heart failure management programme	2006	J Cardiovasc Med (Hagerstown)	7	8	614-622	Nucifora,G et al	wrong population
Improving care at lower cost for end-stage heart and lung disease: integrating end of life planning with home care	2006	Mo Med	103	2	146-151	Edes,T.E et al	not found
A motivational counseling approach to improving heart failure self-care: mechanisms of effectiveness	2006	J Cardiovasc Nurs	21	3	232-241	Riegel,B et al	wrong population
Randomized controlled trial of telephone case management in Hispanics of Mexican origin with heart failure	2006	J Card Fail	12	3	211-219	Riegel,B et al	wrong population
Home telehealth improves clinical outcomes at lower cost for home healthcare	2006	Telemed J E Health	12	2	128-136	Finkelstein,S.M et al	wrong population
The influences of postdischarge management by nurse practitioners on hospital readmission for heart failure	2006	J Am Acad Nurse Pract	18	4	154-160	Delgado-Passler,P et al	systematic review
Prolonged effects of a home-based intervention in patients with chronic illness	2006	Arch Intern Med	166	6	645-650	Pearson,S et al	wrong population
The impact of nurse-directed patient education on quality of life and functional capacity in people with heart failure	2006	J Am Acad Nurse Pract	18	3	116-123	Kutzleb,J et al	wrong population
Computer-based education for patients with chronic heart failure. A randomised, controlled, multicentre trial of the effects on knowledge, compliance and quality of life	2006	Patient Educ Couns	64	1	128-135	Stromberg,A et al	wrong population

[Outpatient medical and nurse management program in patients with chronic heart failure in a large territorial area in Piedmont. Four years of follow-up]	2005	Ital Heart J Suppl	6	12	812-820	Conte,M.R et al	not found
Benefits of comprehensive inpatient education and discharge planning combined with outpatient support in elderly patients with congestive heart failure	2005	Congest Heart Fail	11	6	315-321	Anderson,C et al	wrong population
Disease management produces limited quality-of-life improvements in patients with congestive heart failure: evidence from a randomized trial in community-dwelling patients	2005	Am J Manag Care	11	11	701-713	Smith,B et al	wrong population
Multidisciplinary and multisetting team management programme in heart failure patients affects hospitalisation and costing	2006	Int J Cardiol	111	3	377-385	Piepoli,M.F et al	wrong population
[Randomised clinical trial to evaluate the efficacy of a multi-factorial intervention to reduce hospitalisation and improve the quality of life of patients with heart failure]	2005	Aten Primaria	36	5	280-283	Brotons,C et al	wrong publication type
Effects of a nurse-based heart failure clinic on drug utilization and admissions in a community hospital setting	2005	Scand Cardiovasc J	39	4	199-205	Andersen,M.K et al	wrong population
Heart failure disease management: implementation and outcomes	2005	Whellan DJ	13	5	231-239	Whellan,D.J	systematic review
Effect of home-based telecardiology on chronic heart failure: costs and outcomes	2005	J Telemed Telecare	11		16-18	Scalvini,S et al	wrong population
Impact of care at a multidisciplinary congestive heart failure clinic: a randomized trial	2005	CMAJ	173	1	40-45	Ducharme,A et al	wrong population
Metaanalysis and review of heart failure disease management randomized controlled clinical trials	2005	Am Heart J	149	4	722-729	Whellan,D.J et al	systematic review
Just-in-time evidence-based e-mail "reminders" in home health care: impact on patient outcomes	2005	Health Serv Res	40	3	865-885	Feldman,P.H et al	wrong population

Just-in-time evidence-based e-mail "reminders" in home health care: impact on nurse practices	2005	Health Serv Res	40	3	849-864	Murtaugh,C.M et al	wrong population
Systematic review of multidisciplinary interventions in heart failure	2005	Heart	91	7	899-906	Holl and ,R et al	systematic review
Randomized trial of a nurse-administered, telephone-based disease management program for patients with heart failure	2005	J Card Fail	11	5	358-365	Dunagan,W.C et al	wrong population
State of the science: posthospitalization nursing interventions in congestive heart failure	2005	ANS Adv Nurs	28	2	175-190	Hamner,J.B	systematic review
Patient's education by nurse: what we really do achieve?	2005	Eur J Cardiovasc Nurs	4	2	107-111	Gonzalez,B et al	not found
Noninvasive home telemonitoring for patients with heart failure at high risk of recurrent admission and death: the Trans-European Network-Home-Care Management System (TEN-HMS) study	2005	J Am Coll Cardiol	45	10	1654-1664	Clel and ,J.G et al	wrong population
Prospective evaluation of an outpatient heart failure disease management program designed for primary care: the Oregon model	2005	J Card Fail	11	4	293-298	Hershberger,R.E et al	wrong population
Early outcomes of a care coordination-enhanced telehome care program for elderly veterans with chronic heart failure	2005	Telemed J E Health	11	1	20-27	Schofield,R.S et al	wrong population
Randomised controlled trial of cardiac rehabilitation in elderly patients with heart failure	2005	Eur J Heart Fail	7	3	411-417	Austin,J et al	wrong population
Patients with heart failure in primary health care: effects of a nurse-led intervention on health-related quality of life and depression	2005	Eur J Heart Fail	7	3	393-403	Martensson,J et al	wrong population
Effects of a nurse-led, clinic and home-based intervention on recurrent hospital use in chronic heart failure	2005	Eur J Heart Fail	7	3	377-384	Thompson,D.R et al	wrong population
A multicenter disease management program for hospitalized patients with heart failure	2004	J Card Fail	10	6	473-480	Tsuyuki,R.T et al	wrong population

The effects of nursing interventions to enhance mental health and quality of life among individuals with heart failure	2004	Appl Nurs Res	17	4	248-256	Scott,L.D et al	wrong population
Implementation of guidelines for management of heart failure in heart failure clinic: effects beyond pharmacological treatment	2004	Int J Cardiol	97	3	411-416	Lainscak,M	wrong population
Long-term healthcare and cost outcomes of disease management in a large, randomized, community-based population with heart failure	2004	Circulation	110	23	3518-3526	Galbreath,A.D et al	wrong population
Care management for low-risk patients with heart failure: a randomized, controlled trial	2004	Ann Intern Med	141	8	606-613	DeBusk,R.F et al	wrong population
Managing congestive heart failure using home telehealth	2004	Home Healthc Nurse	22	10	719-722	Schneider,N.M	wrong population
Heart failure clinics and outpatient management: review of the evidence and call for quality assurance	2004	Eur Heart J	25	18	1596-1604	Gustafsson,F et al	systematic review
Multidisciplinary strategies for the management of heart failure patients at high risk for admission: a systematic review of randomized trials	2004	J Am Coll Cardiol	44	4	810-819	McAlister,F.A et al	systematic review
Limited long term effects of a management programme for heart failure	2004	Heart	90	9	1010-1015	Mejhert,M et al	wrong population
A literature review of cardiovascular disease management programs in managed care populations	2004	J Manag Care Pharm	10	4	326-344	Ara,S	systematic review
A randomized intervention to improve heart failure outcomes in community-based home health care	2004	Home Health Care Serv Q	23	1	1-23	Feldman,P.H et al	wrong population
[Case management for patients with congestive heart failure under ambulatory care--a critical review]	2004	Z Arztl Fortbild Qualitatssich	98	2	143-154	Gensichen,J et al	not found
Transitional care of older adults hospitalized with heart failure: a randomized, controlled trial	2004	J Am Geriatr Soc	52	5	675-684	Naylor,M.D et al	wrong population
The comprehensive care team: a controlled trial of outpatient palliative medicine consultation	2004	Arch Intern Med	164	1	83-91	Rabow,M.W et al	wrong population
Effects of education, self-care instruction and physical exercise on patients with chronic heart failure	2003	Z Kardiol	92	12	985-993	Miche,E et al	wrong population

A disease management program for heart failure: collaboration between a home care agency and a care management organization	2003	Lippincotts Case Manag	8	6	265-273	Gorski,L.A et al	wrong population
Palliative care management: a Veterans Administration demonstration project	2003	J Palliat Med	6	5	831-839	Rosenfeld,K et al	background article
A systematic review of telemonitoring for the management of heart failure	2003	Eur J Heart Fail	5	5	583-590	Louis,A.A et al	systematic review
Use of the short form 36 in a primary care based disease management program for patients with congestive heart failure	2003	Dis Manag	6	2	111-117	Sidorov,J et al	wrong population
[Management programs for elderly patients with chronic heart failure]	2003	Clin Ter	154	3	199-206	Pulignano,G et al	not found
Cardiac rehabilitation with nurse care management and telephonic interactions at a community hospital: program evaluation of participation and lipid outcomes	2003	Lippincotts Case Manag	8	4	141-157	Harris,D.E et al	wrong population
Effects of an exercise adherence intervention on outcomes in patients with heart failure	2003	Rehabil Nurs	28	4	117-122	Duncan,K et al	wrong population
Uptake of self-management strategies in a heart failure management programme	2003	Eur J Heart Fail	5	3	371-380	Wright,S.P et al	wrong population
A randomized trial of telenursing to reduce hospitalization for heart failure: patient-centered outcomes and nursing indicators	2003	Home Health Care Serv Q	22	1	1-20	Jerant,A.F et al	wrong population
Case management in a heterogeneous congestive heart failure population: a randomized controlled trial	2003	Arch Intern Med	163	7	809-817	Laramee,A.S et al	wrong population
Heart failure disease management: impact on hospital care, length of stay, and reimbursement	2003	Congest Heart Fail	9	2	77-83	Discher,C.L et al	wrong population
Managing heart failure in the community; role of the nurse specialist	2001	Health Bull (Edinb)	59	5	340-342	Smith,M et al	not found
Outcomes of chronic heart failure	2003	Arch Intern Med	163	3	347-352	Benatar,D et al	wrong population

Telemanagement of heart failure: a diuretic treatment algorithm for advanced practice nurses	2002	Heart Lung	31	5	340-347	Mueller,T.M et al	background article
Symptom status and quality-of-life outcomes of home-based disease management program for heart failure patients	2002	Outcomes Manag	6	4	161-168	Todero,C.M et al	wrong population
Cost/utility ratio in chronic heart failure: comparison between heart failure management program delivered by day-hospital and usual care	2002	J Am Coll Cardiol	40	7	1259-1266	Capomolla,S et al	wrong population
Heart failure management: multidisciplinary care has intrinsic benefit above the optimization of medical care	2002	J Card Fail	8	3	142-148	McDonald,K et al	wrong population
Home-based intervention in congestive heart failure: long-term implications on readmission and survival	2002	Circulation	105	24	2861-2866	Stewart,S et al	wrong population
[Impact of a nurse-based heart failure management program on hospitalization rate, functional status, quality of life, and medical costs]	2002	Ital Heart J Suppl	3	5	532-538	Chinaglia,A et al	not found
Effect of a heart failure clinic on survival and hospital readmission in patients discharged from acute hospital care	2002	Eur J Heart Fail	4	3	353-359	Azevedo,A et al	wrong population
Detecting early clinical deterioration in chronic heart failure patients post-acute hospitalisation-a critical component of multidisciplinary, home-based intervention?	2002	Eur J Heart Fail	4	3	345-351	Stewart,S et al	wrong population
Quantification of interventions and outcomes in an outpatient telemanagement and care management congestive heart failure program	2000	Congest Heart Fail	6	3	146-151	Palladino,M et al	wrong population
Quality of life of individuals with heart failure: a randomized trial of the effectiveness of two models of hospital-to-home transition	2002	Med Care	40	4	271-282	Harrison,M. et al	wrong population
APN spells success for a heart failure program	2002	Nurs Manage	33	2	46-48	Dahl,J et al	background article
Improving the quality of home care for patients with heart failure	2002	Caring	21	3	10-14	Gorski,L.A	not found

Effect of a standardized nurse case-management telephone intervention on resource use in patients with chronic heart failure	2002	Arch Intern Med	162	6	705-712	Riegel,B et al	wrong population
Use of a clinical care map for the management of congestive heart failure in a community hospital	2001	Congest Heart Fail	7	1	37-42	Lanzieri,M et al	wrong population
A randomized trial of the efficacy of multidisciplinary care in heart failure outpatients at high risk of hospital readmission	2002	J Am Coll Cardiol	39	3	471-480	Kasper,E.K et al	wrong population
Randomized, controlled trial of integrated heart failure management: The Auckland Heart Failure Management Study	2002	Eur Heart J	23	2	139-146	Doughty,R.N et al	wrong intervention
Compliance and effectiveness of 1 year's home telemonitoring. The report of a pilot study of patients with chronic heart failure	2001	Eur J Heart Fail	3	6	723-730	de,Lusignan S et al	wrong population
Reducing the cost of frequent hospital admissions for congestive heart failure: a randomized trial of a home telecare intervention	2001	Med Care	39	11	1234-1245	Jerant,A et al	wrong population
Cost-effective outpatient management of persons with heart failure	2001	Prog Cardiovasc Nurs	16	2	50-56	Henrick,A	wrong population
A systematic review of randomized trials of disease management programs in heart failure	2001	Am J Med	110	5	378-384	McAlister,F.A et al	systematic review
Effectiveness of team-managed home-based primary care: a randomized multicenter trial	2000	JAMA	284	22	2877-2885	Hughes,S.L et al	wrong population
Which patients with heart failure respond best to multidisciplinary disease management?	2000	J Card Fail	6	4	290-299	Riegel,B et al	wrong population
A pilot study of radiotelemetry for continuous cardiopulmonary monitoring of patients at home	2000	J Telemed Telecare	6		S119-S122	de,Lusignan S et al	wrong population
A controlled pilot study in the use of telemedicine in the community on the management of heart failure--a report of the first three months	1999	Stud Health Technol Inform	64		126-137	de,Lusignan S et al	not found
Impact of a nurse-managed heart failure clinic: a pilot study	2000	Am J Crit Care	9	2	140-146	Paul,S	not found

Enhanced access to primary care for patients with congestive heart failure. Veterans Affairs Cooperative Study Group on Primary Care and Hospital Readmission	1999	Eff Clin Pract	2	5	201-209	Oddone,E.Z et al	wrong population
Implementing a congestive heart failure disease management program to decrease length of stay and cost	1999	J Cardiovasc Nurs	14	1	55-74	Knox,D et al	not found
Effect of a home monitoring system on hospitalization and resource use for patients with heart failure	1999	Am Heart J	138	4	633-640	Heidenreich,P.A et al	wrong population
A community hospital-based congestive heart failure program: impact on length of stay, admission and readmission rates, and cost	1999	Am J Manag Care	5	1	37-43	Rauh,R.A et al	wrong population
Heart failure disease management: a critical review	1999	J Card Fail	5	1	64-75	Rich,M.W	systematic review
Comprehensive multidisciplinary programs for the management of patients with congestive heart failure	1999	J Gen Intern Med	14	2	130-135	Philbin,E.F	systematic review
Cost effective management programme for heart failure reduces hospitalisation	1998	Heart	80	5	442-446	Cline,C.M et al	wrong population
Effects of a home-based intervention among patients with congestive heart failure discharged from acute hospital care	1998	Arch Intern Med	158	10	1067-1072	Stewart,S et al	wrong population
Using case management with clinical plans to improve patient outcomes	1998	Home Healthc Nurse	16	1	15-20	Huggins,C.M et al	background article
Symptomatic improvement and reduced hospitalization for patients attending a cardiomyopathy clinic	1997	Clin Cardiol	20	11	949-954	Smith,L.E et al	wrong population
Assessment--patients, chronic heart failure, and home care	1997	Caring	16	6	20-2, 24	Lazarre,M et al	not found
A study of the relationship between home care services and hospital readmission of patients with congestive heart failure	1997	Home Healthc Nurse	15	2	123-129	Martens,K.H et al	wrong population

A comprehensive management system for heart failure improves clinical outcomes and reduces medical resource utilization	1997	Am J Cardiol	79	1	58-63	West,J.A et al	wrong population
Disease management for chronic congestive heart failure	1996	J Cardiovasc Nurs	11	1	54-62	Brass-Mynderse,N.J	background article
Does increased access to primary care reduce hospital readmissions? Veterans Affairs Cooperative Study Group on Primary Care and Hospital Readmission	1996	N Engl J Med	334	22	1441-1447	Weinberger,M et al	wrong population
The effect of a nurse-managed CHF clinic on patient readmission and length of stay	1996	Home Healthc Nurse	14	5	351-356	Lasater,M.	background article
The connection delivery model: care across the continuum	1996	Nurs Manage	27	5	34, 36	Donlevy,J.A et al	not found
A multidisciplinary intervention to prevent the readmission of elderly patients with congestive heart failure	1995	N Engl J Med	333	18	1190-1195	Rich,M.W et al	wrong population
Home monitoring for congestive heart failure patients	1995	Caring	14	8	53-54	Miller,P.Z	wrong population
Prevention of readmission in elderly patients with congestive heart failure: results of a prospective, randomized pilot study	1993	J Gen Intern Med	8	11	585-590	Rich,M.W et al	wrong population
Improving Heart Failure Self-Management Support by Actively Engaging Out-of-Home Caregivers: Results of a Feasibility Study	2013	Congest Heart Fail	14	1		Piette,J.D et al	wrong population
Blended collaborative care to treat heart failure and comorbid depression: Rationale and Study Design of the Hopeful Heart Trial	2019	Psychosom Med	81	6	495-505	Herbeck,Belnap B et al	wrong population
Application of transitional care model in patients with chronic heart disease: A case-controlled intervention study	2018	Rev Latam HTA	13	3	285-290	Rezapour-Nasrabad,R	wrong population
Clinical investigation on the nursing needs of heart failure patients and analysis of planning nursing intervention effect	2017	Biomedical Research	28	22	10084-10087	Chen,C et al	wrong population

Palliative Care Interventions for Patients with Heart Failure: A Systematic Review and Meta-Analysis	2017	J Palliat Med	20	1	84-92	Diop,M.S et al	systematic review
Case conference primary-secondary care planning at end of life can reduce the cost of hospitalisations	2016	BMC Palliat Care	15	1		Hollingworth,S et al	wrong population
Primary results of the Patient-Centered Disease Management (PCDM) for heart failure study a randomized clinical trial	2015	JAMA Intern Med	175	5	725-732	Bekelman,D.B et al	wrong population
A Mobile Health Intervention Supporting Heart Failure Patients and Their Informal Caregivers: A Randomized Comparative Effectiveness Trial	2015	J Med Internet Res	17	6	e142	Piette,J.D et al	wrong population
Program evaluation of remote heart failure monitoring: healthcare utilization analysis in a rural regional medical center	2015	Telemed J E Health	21	3	157-162	Riley,W.T et al	wrong population
Cardiauvergne: Heart failure home management and remote monitoring system. [French]	2014	Soins	3	4	169-176	Eschalier,R et al	wrong population
Manpower and outpatient clinic workload for remote monitoring of patients with cardiac implantable electronic devices: Data from the HomeGuide registry	2014	J Cardiovasc Electrophysiol	25	11	1216-1223	Ricci,R.P et al	wrong population
Home-based advance care programme is effective in reducing hospitalisations of advanced heart failure patients: A clinical and healthcare cost study	2013	Ann Acad Med Singap	42	9	466-471	Wong,R.C.C et al	not found
Remote monitoring after recent hospital discharge in patients with heart failure: A systematic review and network meta-analysis	2013	Heart	99	23	1717-1726	Pandor, A et al	systematic review
Mode of action and effects of standardized collaborative disease management on mortality and morbidity in patients with systolic heart failure the interdisciplinary network for heart failure (INH) study	2012	Circ Heart Fail	5	1	25-35	Angermann,C.E et al	wrong population

Outcomes of a telehealth intervention for homebound older adults with heart or chronic respiratory failure: a randomized controlled trial	2012	Gerontologist	52	4	541-552	Gellis,Z.D et al	wrong population
Hospital initiative reduces heart failure readmissions	2012	Hosp Case Manag	20	11	161-163	No authors listed	not found
The "Kremser model": Successful disease management programme for patients with chronic heart failure. [German]	2011	Journal fur Kardiologie	18	9	299-306	Bohmer,A et al	wrong population
Telemonitoring with case management for seniors with heart failure	2011	Am J Manag Care	17	3	e71-e79	Wade,M.J et al	wrong population
Clinical trials update from the European Society of Cardiology Heart Failure meeting 2011: TEHAF, WHICH, CARVIVA, and atrial fibrillation in GISSI-HF and EMPHASIS-HF	2011	Eur J Heart Fail	13	10	1147-1151	Clel and ,J.G.F et al	wrong population
Improving heart failure in home care with chronic disease management and telemonitoring	2010	Home Healthc Nurse	28	10	606-617	Hall,P et al	background article
The effect of shared medical visits on knowledge and self-care in patients with heart failure: A pilot study	2009	Heart Lung	38	1	25-33	Yehle,K.S et al	wrong population
Telehealth helps hospital cut readmissions by 75%	2007	Healthcare Benchmarks Qual Improv	14	8	92-94	No authors listed	not found
Therapeutic education and multidisciplinary approaches in heart failure. [French]	2009	Medecine Therapeutique - Cardio	4	1	79-87	Assyag,P et al	not found
Community cardiology clinics for secondary prevention of coronary heart disease and heart failure in primary care	2008	Cardiol Review	25	12	64-67	Khunti,K et al	not found
Impact of specialist care in patients with newly diagnosed heart failure: A randomised controlled study	2007	Int J Cardiol	115	2	196-202	Rao,A et al	wrong population
Feasibility and efficacy of a hybrid post-discharge service for patients with acute heart failure - The tyrolean model. [German]	2007	Journal fur Kardiologie	14	1	13-17	Metzler,B et al	not found

Alternative healthcare models for patients with heart failure: A systematic review. [Spanish]	2006	Revista de Calidad Asistencial	21	1	51-60	Morales,J.M et al	systematic review
Tele-homecare for chronically-ill patients: Improved outcomes and new developments	2004	Journal on Information Technology in Healthcare	2	4	251-262	Robinson,S et al	wrong population
Management of the Patient with Congestive Heart Failure in the Home Care and Palliative Care Setting	2004	Annals of Long-Term Care	12	1	33-37	Quaglietti,S et al	not found
A community-based service for patients with congestive cardiac failure: Impact on quality of life scores	2004	British Journal of Cardiology	11	1	75-79	Williams,H et al	not found
Interdisciplinary team-based management of heart failure	2003	Disease Management and Health Outcomes	11	2	87-94	Di Salvo,T.G et al	background article
Care management interventions for older patients with congestive heart failure	2003	American Journal of Managed Care	9	6	447-459	Windham,B.G et al	not found
Establishment of a nurse-led heart failure clinic. Design and baseline data from the first two years. [Danish]	2003	Ugeskrift for Laeger	165	7	686-690	Galatius,S et al	not found
Specialist Nurse-Led Intervention in Outpatients with Congestive Heart Failure: Impact on Clinical and Economic Outcomes	2003	Disease Management and Health Outcomes	11	11	693-698	Palmer,N.D et al	background article
Heart failure programs and comprehensive management in heart failure. [Italian]	2002	Monaldi Archives for Chest Disease	58	2	135-139	Di,Lenarda A et al	background article
Standardized telephonic case management in a Hispanic heart failure population: An effective intervention	2002	Disease Management and Health Outcomes	10	4	241-249	Riegel,B et al	wrong population
An economic analysis of specialist heart failure nurse management in the U.K.: Can we afford not to implement it?	2002	European Heart Journal	23	17	1369-1378	Stewart,S et al	wrong population
Optimising delivery of care for chronic heart failure	2000	Journal of Clinical Excellence	1	4	209-215	Clark,A.L et al	not found

Cost-effectiveness analysis in clinical practice: The case of heart failure	1999	Archives of Internal Medicine	159	15	1690-1700	Rich,M.W et al	systematic review
Structured telephone support or nonÂƒ__invasive telemonitoring for patients with heart failure	2015	Cochrane Database of Syst Rev		10	CD007228	Inglis,S.C et al	systematic review
A new programme of multidisciplinary care for patients with heart failure in PoznaÂ ": one-year follow-up	2006	Kardiol Pol	64	10	1063-70	Wierzchowiecki, M et al	not found
Efficacy of a Physician-Led Multiparametric Telemonitoring System in Very Old Adults with Heart Failure	2015	J Am Geriatr Soc	63	6	1175-80	Pedone,C et al	wrong population
A randomised controlled trial of a facilitated home-based rehabilitation intervention in patients with heart failure with preserved ejection fraction and their caregivers: the REACH-HFpEF Pilot Study	2018	BMJ Open	8	4	e019649	Lang,C.C et al	wrong population
Evaluation of a home-based intervention in heart failure patients. Results of a randomized study	2005	Rev Esp Cardiol	58	6	618-25	Morcillo,C et al	wrong population
Economic evaluation of Manitoba Health Lines in the management of congestive heart failure	2013	Healthc Policy	9	2	36-50	Cui,Y et al	wrong population
Physical function and quality of life in older women with diastolic heart failure: effects of a progressive walking program on sleep patterns	2007	Prog Cardiovasc Nurs	22	2	72-80	Gary,R et al	wrong population
Randomized, controlled evaluation of short- and long-term benefits of heart failure disease management within a diverse provider network: the SPAN-CHF trial	2004	Circulation	110	11	1450-5	Kimmelstiel,C et al	wrong population
Cognitive Behavior Therapy for Depression and Self-Care in Heart Failure Patients: a Randomized Clinical Trial	2015	JAMA Intern Med	175	11	1773-82	Freedl and ,K.E et al	wrong population
Outcomes of home management methods for chronic heart failure	2002	Doctoral thesis				Bondmass,M.D	not found

Effects of three different disease management programs on outcomes in patients hospitalized with heart failure: a randomized trial	2012	Med Clin (Barc)	138	5	192-8	Gamez-Lopez,A.L et al	wrong population
A randomized controlled trial of self-management programme improves health-related outcomes of older people with heart failure	2013	J Adv Nurs	69	11	2458-69	Jung-Hua, S et al	wrong population
A Review: Discharge Navigation and Its Effect on Heart Failure Readmissions	2014	Prof Case Manag	19	5	224-234	Schell,W	systematic review
Advanced Practice Nurse Intervention and Heart Failure Readmissions	2018	Doctoral thesis				Reynolds,T	wrong population
Case management for patients with heart failure: a quality improvement intervention	2005	J Gerontol Nurs	31	5	20-28	Miller,L.C et al	not found
Chronic Heart Failure Management in Rural Primary Care	2016	J Dr Nurs Pract	9	1	20-28	Kreifels,E et al	not found
Clinical pathway versus a usual plan of care for patients with congestive heart failure: what's the difference?...part 1 of a two-part series	2001	Home Healthc Nurse	19	3	142-150	Hoskins,L.M et al	wrong population
Communication Between the Primary Care Physician and the Hospitalist at theTime of Patient Admission	2012	JCOM	19	10	453-459	Hennrikus,E	wrong population
Comparison of Health Buddy with traditional approaches to heart failure management	2003	Fam Community Health	26	4	275-288	LaFramboise,L.M et al	wrong population
Effectiveness of Interprofessional Care Teams on Reducing Hospital Readmissions in Patients with Heart Failure: A Systematic Review	2018	MedSurg Nursing	27	3	177-185	Shah,B	systematic review
Effects of education on self-care behaviour and quality of life in patients with chronic heart failure	2010	The World of Critial Care Nursing	7	2	115-121	Enç, N et al	wrong population
Effects of home-based care program on symptom alleviation and well-being among persons with chronic heart failure	2008	Thai Journal of Nursing Research	12	1	25-39	Wongpiriyayothar ,A et al	not found
Implementing a congestive heart failure outpatient program	1999	not listed	6	7	14-18	Schwabauer,N.J et al	not found

Incorporating Technology to Decrease Heart Failure Readmission Rates	2018	Doctoral thesis				Thames,V	wrong population
Nursing approach to a patient with cardiac insufficiency in Primary Care	2012	Metas de enfemería	15	10	56-61	Prats-Guardiola, M et al	not found
Randomized control trial of a self-management intervention for heart failure older adults in Northern Taiwan	2019	Australian Collage of Nursing	26	2	288-294	Shao,J et al	wrong population
Research corner. Utilization of home health care services by elderly patients with heart failure	1998	not listed	10	4	66-73	Moulton,P.J et al	wrong population
Research on reducing hospitalizations in patients with chronic heart failure	2010	Home Healthc Nurse	28	6	335-340	McGhee,G et al	background article
Self-care and improved outcomes: an intervention by heart failure nurse specialists	2016	British J Cardiac Nurs	11	2	82-88	MacInnes,J et al	wrong population
Technology-enhanced practice for patients with chronic cardiac disease: Home implementation and evaluation	2010	Heart Lung	39	6	S34-S46	Brennan,P.F et al	wrong population
Telephone-based, nursing follow-up of patients with heart failure	2011	not listed	14	3	22-26	Just,M et al	wrong population
The Influence of Remote Monitoring on Clinical Decision Making	2016	Home Healthc Manag Prac	28	2	86-93	Williams,C et al	not found
The role of community-based nursing interventions in improving outcomes for individuals with cardiovascular disease: A systematic review	2019	Int J Nurs Stud	100		N-N	Han,E et al	systematic review
The impact of a community-based heart failure multidisciplinary team clinic on healthcare utilization and costs	2022	ESC Heart Fail.	9	1	676–684	Weinstein et al	wrong intervention
Examining Adherence and Dose Effect of an Early Palliative Care Intervention for Advanced Heart Failure Patients	2021	J Pain Symptom Manage.	62	3	471-481	Wells, R	wrong study design
Association between communitybased nurse practitioner support, self-care behaviour and quality of life in patients with chronic heart failure	2021	Australian Journal of Advanced Nursing	38	3	25-32	Sheau Huey, Chen	wrong population

An inpatient multidisciplinary educational approach to reduce 30-day heart failure readmissions	2021	Saudi Pharm J	29	4	337-342	Aljabri, A	wrong population
A Randomized Controlled Trial of Heart Failure Disease Management in Skilled Nursing Facilities	2022	J Am Med Dir Assoc	23	3	359-366	Boxer, R. S. et al	wrong population
Challenges of Telemonitoring Programs for Complex Chronic Conditions: Randomized Controlled Trial With an Embedded Qualitative Study	2022	J Med Internet Res	24	1	e31754	Ware, P et al	wrong population
Disease Management in Skilled Nursing Facilities Improves Outcomes for Patients With a Primary Diagnosis of Heart Failure	2021	Journal of the American Medical Directors Association	0	0	367-372	Weerahandi, H et al	wrong population
Disease management with home telemonitoring aimed at substitution of usual care in the Netherlands: Post-hoc analyses of the e-Vita HF study	2022	J Cardiol	79	1	1-5	Brons, M et al	wrong population
Efficacy of Blended Collaborative Care for Patients With Heart Failure and Comorbid Depression: A Randomized Clinical Trial	2021	JAMA Intern Med	181	10	1369-1380	Rollman, B. L et al	wrong population
Implementation of early follow-up care after heart failure hospitalization	2021	Am J Manag Care	27	2	E42-e47	Dev, S et al	wrong population
Managing patients with heart failure: contemporary real-world experience	2022	BMC research notes	15	1	41	Siddiqui, M et al	wrong population
The effectiveness of a nurse-led home-based heart failure self-management programme (the HOM-HEMP) for patients with chronic heart failure: A three-arm stratified randomized controlled trial	2021	Int J Nurs Stud	122	0	104026	Jiang, Y et al	wrong population
The Effect of Rehospitalization and Emergency Department Visits on Subsequent Adherence to Weight Telemonitoring	2021	J Cardiovasc Nurs	36	5	482-488	Haynes, S. C et al	wrong population
The Impact of Nurse-Led Cardiac Rehabilitation on Quality of Life and Biophysiological Parameters in Patients With Heart Failure: A Randomized Clinical Trial	2021	Journal of Nursing Research	29	1	e130-e130	Arjunan et al	wrong population

A quasi-experimental study examining a nurse-led educational program to improve disease knowledge and self-care for patients with acute decompensated heart failure with reduced ejection fraction	2021	Adv Clin Exp Med	0	0	-	Kolasa, J et al	wrong population
Effect of nurse-led hospital-to-home transitional care interventions on mortality and psychosocial outcomes in adults with heart failure: a meta-analysis	2021	Eur J Cardiovasc Nurs	18	0	zvab105	Li, Y et al	systematic review
The effectiveness of transitional care interventions for adult people with heart failure on patient-centered health outcomes: A systematic review and meta-analysis including dose-response relationship	2021	International journal of nursing studies	117	0	103902	Li, Y et al	systematic review
Effects of nurse-led transitional care interventions for patients with heart failure on healthcare utilization: A meta-analysis of randomized controlled trials	2021	PLoS One	16	12	E0261300	Li, Y et al	systematic review
The impact of nurse practitioners on health outcomes in outpatient heart failure management: a systematic review	2021	Canadian Journal of Cardiovascular Nursing	31	2	13-17	Fichadiya et al	systematic review
The impact of nurse-led community-based models of care on hospital admission rates in heart failure patients: An integrative review	2021	Heart Lung	50	5	685-692	Ledwin et al	systematic review

Table S2. ROBINS-I adaptation

Bias domain	Signaling questions	Description	Response option
Domain 1. Bias due to confounding (prognostic factors - thus related to the outcome- also related to intervention assignment)	1.1 Is there any reason to think that patients were "too much stable" during the period? or patients were too decompensate during the period?	The first question assesses the risk of the survivor, the second assesses the risk of the disease's own decline	Y/PY/PN/N
	1.4 If Y or PY to 1.1: The authors performed any specific analysis to mitigate that bias?		Y/PY/PN/N
	1.6. Did the authors control for any post-intervention variables that could have been affected by the intervention?	Controlling for post-intervention variables that are affected by intervention is not appropriate. Controlling for mediating variables estimates the direct effect of intervention and may introduce bias. Controlling for common effects of intervention and outcome introduces bias.	Y/PY/PN/N
			Risk of bias:
Domain 2. Bias in selection of participants into the study	2.1 Was selection of participants into the study (or into the analysis) based on participant characteristics observed after the start of intervention?	Inclusion criteria previously defined	Y/PY/PN/N
	2.4 Do start of follow-up and start of intervention coincides for most participants?	If participants are not followed from the start of the intervention then a period of follow up has been excluded, and individuals who experienced the outcome soon after intervention will be missing from analyses. This problem may occur when prevalent, rather than	Y/PY/PN/N

		new (incident), users of the intervention are included in analyses.	
	2.5 If N/PN to 2.4:<br><br>Were adjustment techniques used that are likely to correct for the presence of selection biases?	It is in principle possible to correct for selection biases, for example by using inverse probability weights to create a pseudo-population in which the selection bias has been removed, or by modelling the distributions of the missing participants or follow up times and outcome events and including them using missing data methodology. However such methods are rarely used and the answer to this question will usually be "No".	Y/PY/PN/N
			Risk of bias:
Domain 3. Bias in classification of interventions	3.1 There was a previous clearly defined intervention?	Judge whether the intervention is sufficiently defined	Y/PY/PN/N
	3.2 The inclusion and exclusion criteria for participants were clearly defined?	Judge if inclusion and exclusion criteria are sufficiently defined	Y/PY/PN/N
	3.3 Could classification of intervention status have been affected by knowledge of the outcome or risk of the outcome?		Y/PY/PN/N
			Risk of bias:
Domain 4. Bias due to deviations from intended interventions	4.1 Were there deviations from the intended intervention beyond what would be expected in usual practice?	Deviations that happen in usual practice following the intervention (for example, cessation of a drug intervention because of acute toxicity) are part of the intended intervention and therefore do not lead to bias in the effect of assignment to intervention.	Y/PY/PN/N

	4.2 If Y/PY to 4.1: Were these deviations balanced in all the intervention group?	If deviations appeared, were homogeneous for all the intervention group?	Y/PY/PN/N
			Risk of bias:
Domain 5. Bias due to missing data	5.1 Were outcome data available for all, or nearly all, participants?	"Nearly all" should be interpreted as "enough to be confident of the findings", and a suitable proportion depends on the context. In some situations, availability of data from 95% (or possibly 90%) of the participants may be sufficient, providing that events of interest are reasonably common in both intervention groups. One aspect of this is that review authors would ideally try and locate an analysis plan for the study.	Y/PY/PN/N
	5.2 Were participants excluded due to missing data on intervention status?	Missing intervention status may be a problem. This requires that the intended study sample is clear, which it may not be in practice.	Y/PY/PN/N
	5.3 Were participants excluded due to missing data on other variables needed for the analysis?	This question relates particularly to participants excluded from the analysis because of missing information on confounders that were controlled for in the analysis.	Y/PY/PN/N
	5.5 If PN/N to 5.1, or Y/PY to 5.2 or 5.3: Is there evidence that results were robust to the presence of missing data?	Evidence for robustness may come from how missing data were handled in the analysis and whether sensitivity analyses were performed by the investigators, or occasionally from additional analyses performed by the systematic reviewers. It is important to assess whether assumptions employed in analyses are clear and plausible. Both content knowledge and statistical expertise will often be required for this. For instance, use of a statistical method such as multiple	Y/PY/PN/N

		imputation does not guarantee an appropriate answer. Review authors should seek naïve (complete-case) analyses for comparison, and clear differences between complete-case and multiple imputation-based findings should lead to careful assessment of the validity of the methods used.	
			Risk of bias:
Domain 6. Bias in measurement of outcomes	6.1 Could the outcome measure have been influenced by knowledge of the intervention received?	Some outcome measures involve negligible assessor judgment, e.g. all-cause mortality or non-repeatable automated laboratory assessments. Risk of bias due to measurement of these outcomes would be expected to be low.	Y/PY/PN/N
	6.2 Were outcome assessors aware of the intervention received by study participants?	If outcome assessors were blinded to intervention status, the answer to this question would be 'No'. In other situations, outcome assessors may be unaware of the interventions being received by participants despite there being no active blinding by the study investigators; the answer this question would then also be 'No'. In studies where participants report their outcomes themselves, for example in a questionnaire, the outcome assessor is the study participant. In an observational study, the answer to this question will usually be 'Yes' when the participants report their outcomes themselves.	Y/PY/PN/N
	6.3 Were comparable methods before and after the intervention?	Comparable assessment methods (i.e. data collection) would involve the same outcome detection methods and thresholds, same time point, same definition, and same measurements.	Y/PY/PN/N

	6.4 Were any systematic errors in measurement of the outcome related to intervention received?	This question refers to differential misclassification of outcomes. Systematic errors in measuring the outcome, if present, could cause bias if they are related to intervention or to a confounder of the intervention-outcome relationship. This will usually be due either to outcome assessors being aware of the intervention received or to non-comparability of outcome assessment methods, but there are examples of differential misclassification arising despite these controls being in place.	Y/PY/PN/N
			Risk of bias:
Domain 7. Bias in selection of the reported result	Is the reported effect estimate likely to be selected, on the basis of the results, from...<br>7.1. ... multiple outcome measurements within the outcome domain?	For a specified outcome domain, it is possible to generate multiple effect estimates for different measurements. If multiple measurements were made, but only one or a subset is reported, there is a risk of selective reporting on the basis of results.	Y/PY/PN/N
	7.2 ... multiple analyses of the intervention outcome relationship?	Because of the limitations of using data from non-randomized studies for analyses of effectiveness (need to control confounding, substantial missing data, etc), analysts may implement different analytic methods to address these limitations. Examples include unadjusted and adjusted models; use of final value vs change from baseline vs analysis of covariance; different transformations of variables; a continuously scaled outcome converted to categorical data with different cut-points; different sets of covariates used for adjustment; and different analytic strategies for dealing with missing data. Application of such methods	Y/PY/PN/N

		generates multiple estimates of the effect of the intervention versus the comparator on the outcome. If the analyst does not pre-specify the methods to be applied, and multiple estimates are generated but only one or a subset is reported, there is a risk of selective reporting on the basis of results.	
	7.3 ... different subgroups?	Particularly with large cohorts often available from routine data sources, it is possible to generate multiple effect estimates for different subgroups or simply to omit varying proportions of the original cohort. If multiple estimates are generated but only one or a subset is reported, there is a risk of selective reporting on the basis of results.	Y/PY/PN/N
			Risk of bias:

Table S3. Assessment of Risk of bias (RoB) in Randomized controlled trial studies.

Outcome assessment: All-cause mortality

	Random sequence generation (selection bias)	Deviations from the intented intervention	Incomplete outcome data (attrition bias)	Bias measuring the outome	Selection reporting result (reporting bias)
De la Porte 2007	?	?	+	+	?
Ekman 1998	+	+	+	+	+
Goldberg 2003	−	?	+	+	?
Lynga 2012	−	+	+	+	?
McDonald 2001	−	−	−	−	?
Rogers 2017	?	+	+	+	?
Smith 2015	+	+	+	+	+

Outcome assessment: Hospitalizations for heart failure

	Random sequence generation (selection bias)	Deviations from the intented intervention	Incomplete outcome data (attrition bias)	Bias measuring the outome	Selection reporting result (reporting bias)
Boyne 2012	?	+	−	+	+
Comin-Colet 2015	+	+	+	+	?
De la Porte 2007	?	?	−	+	?
Ekman 1998	+	+	+	+	+
GESICA 2005	+	+	+	+	+
Goldberg 2003	−	?	+	+	?
Jaarsma 1999	−	−	+	−	+
Lynga 2012	−	+	+	+	?
McDonald 2001	−	−	−	−	?
Rogers 2017	?	+	−	+	?
Smith 2015	+	+	+	+	+

Outcome assessment: All-cause hospitalizations

	Random sequence generation (selection bias)	Deviations from the intented intervention	Incomplete outcome data (attrition bias)	Bias measuring the outome	Selection reporting result (reporting bias)
Aiken 2006	+	+	−	−	−
Brännström 2014	?	+	?	?	−
Ekman 1998	+	+	+	+	+
Goldberg 2003	−	?	+	+	?
Jaarsma 1999	−	−	+	−	+
Lynga 2012	−	+	+	+	?
Ong 2016	?	+	−	+	+
Yuet 2016	−	+	−	+	?

Outcome assessment: Quality of life

	Random sequence generation (selection bias)	Deviations from the intented intervention	Incomplete outcome data (attrition bias)	Bias measuring the outome	Selection reporting result (reporting bias)
Aiken 2006	+	+	+	−	−
Bondmass 2007	−	+	+	−	?
Brännström 2014	?	+	−	−	−
De la Porte 2007	?	?	−	−	?
Goldberg 2003	−	?	−	−	?
Jaarsma 2000	−	−	+	−	+
Man 2017	+	+	+	−	+
Rogers 2017	?	+	−	−	?
Yuet 2016	−	+	−	−	?

Outcome assessment: Self-care

	Random sequence generation (selection bias)	Deviations from the intented intervention	Incomplete outcome data (attrition bias)	Bias measuring the outome	Selection reporting result (reporting bias)
De la Porte 2007	?	?	−	−	?
Jaarsma 2000	−	−	+	−	+
Zamanzadeh 2013	?	+	+	−	?

Table S4. Assessment of Risk of bias with adaptation ROBINS-I tool in non-Randomized controlled trial studies.

Outcome assessment: Hospitalizations for heart failure

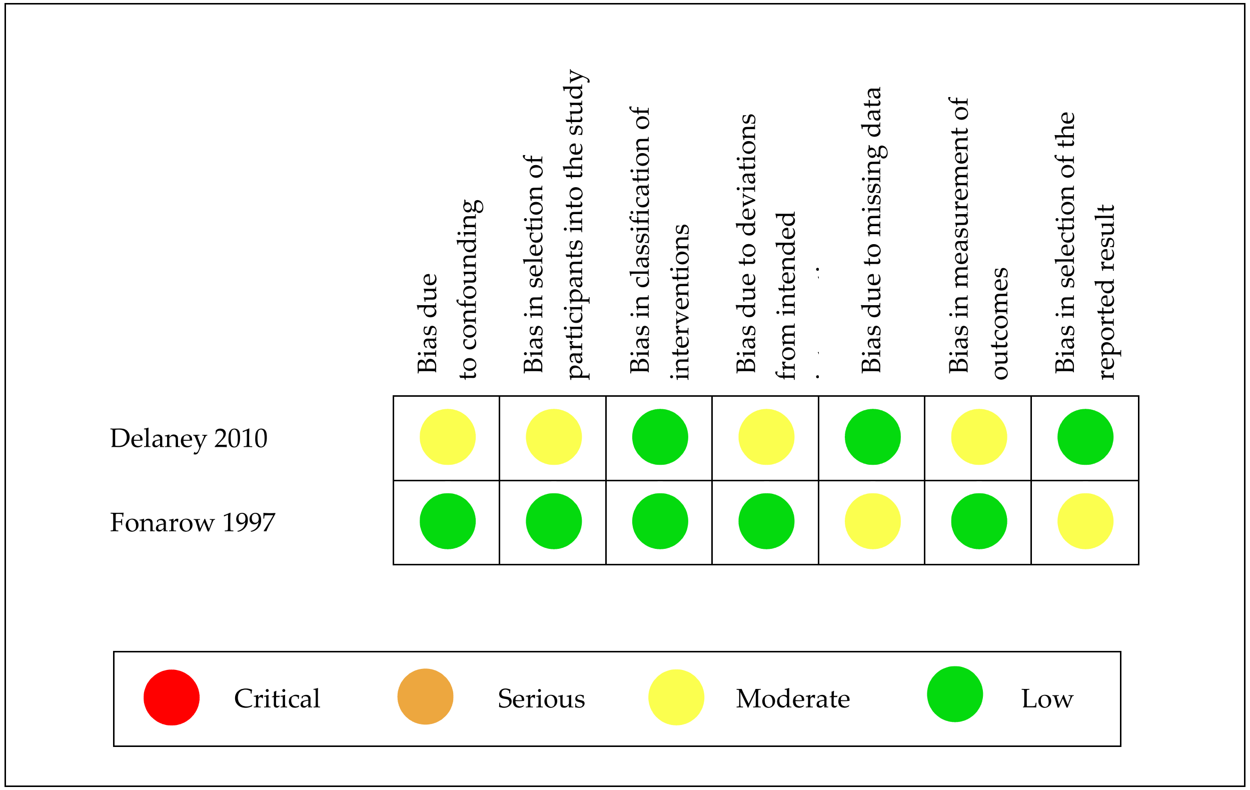

Outcome assessment: All-cause hospitalizations

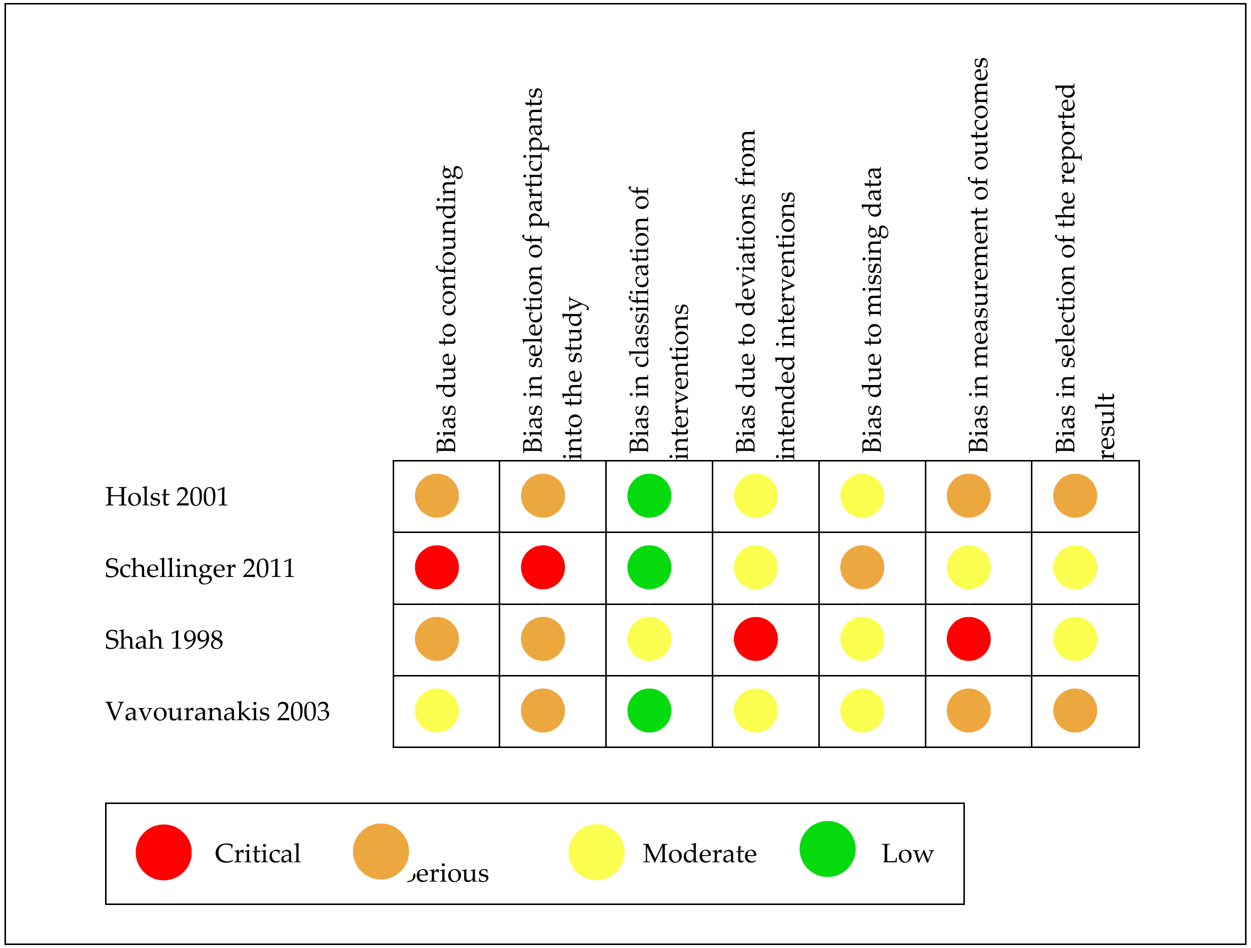

Outcome assessment: Quality of life

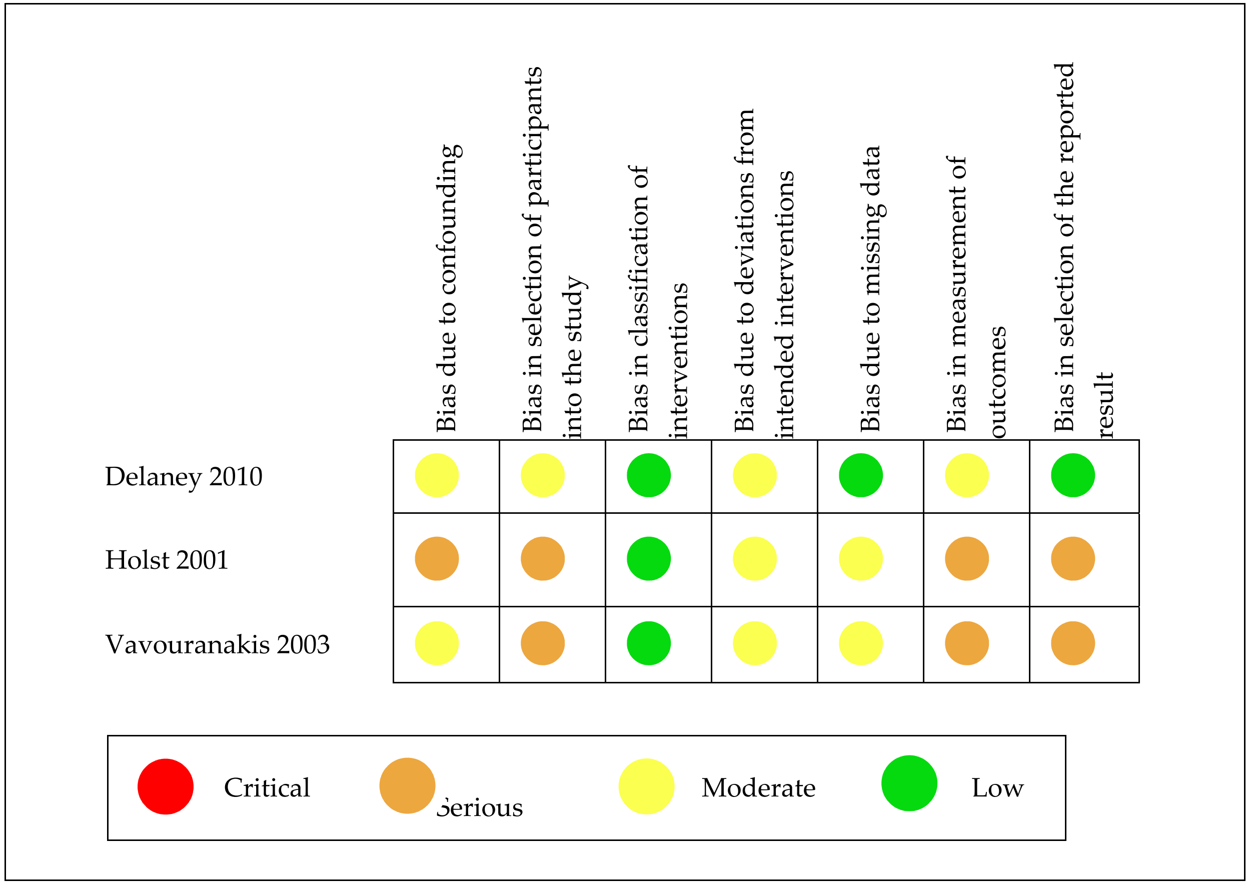

Figure S1. Meta-analysis of subgroups according follow-up time, type of nurse-led case management delivered and age.

All-cause mortality	Follow-up subgroup analysis 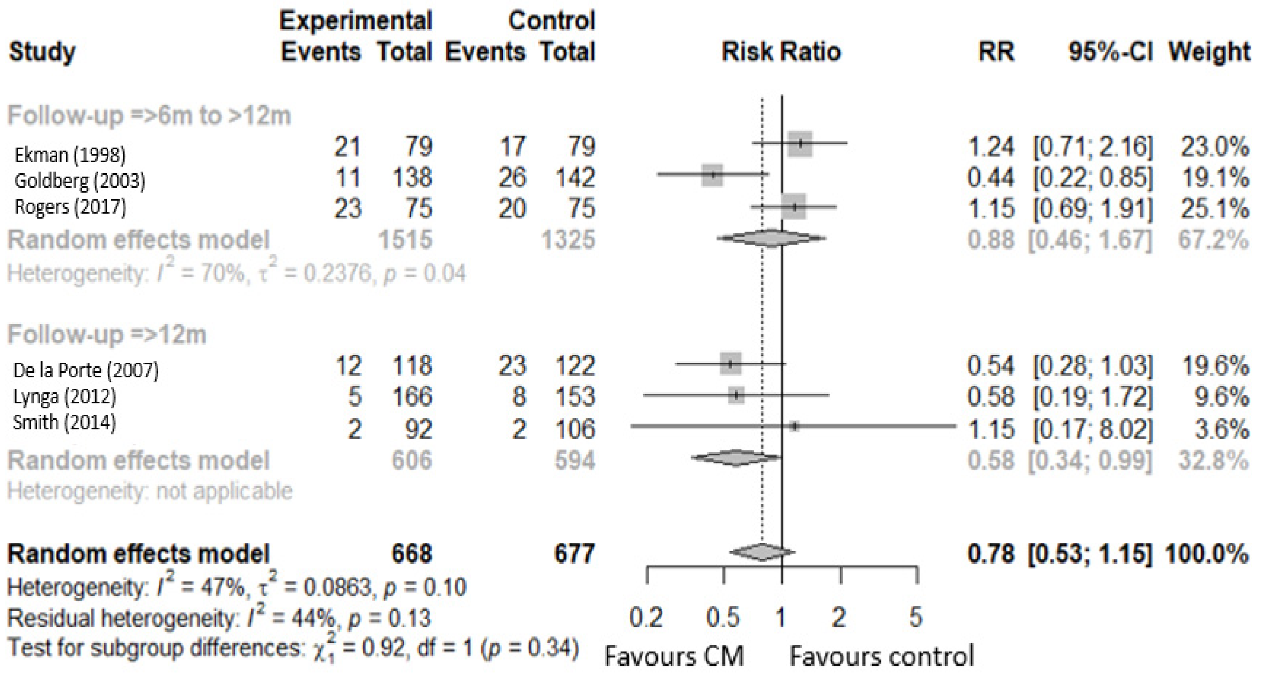

Type of nurse-led case management delivered subgroup analysis

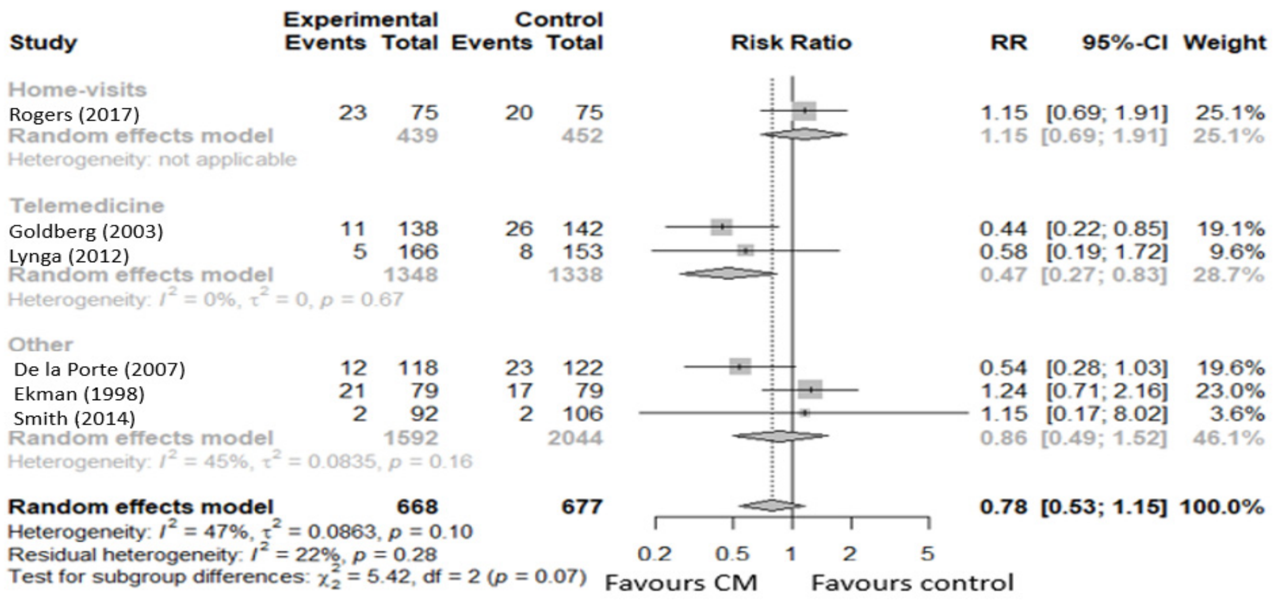

Age subgroup analysis

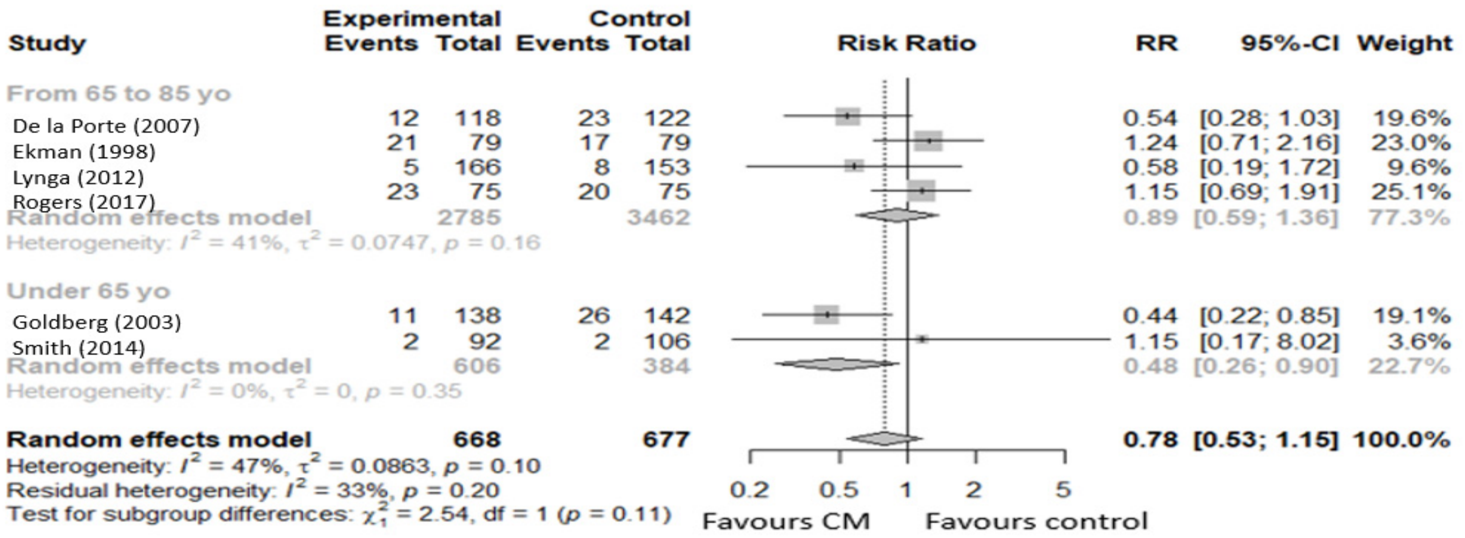

<table>
<tr>
<td>Hospitalizations for heart failure</td>
<td>Follow-up subgroup analysis

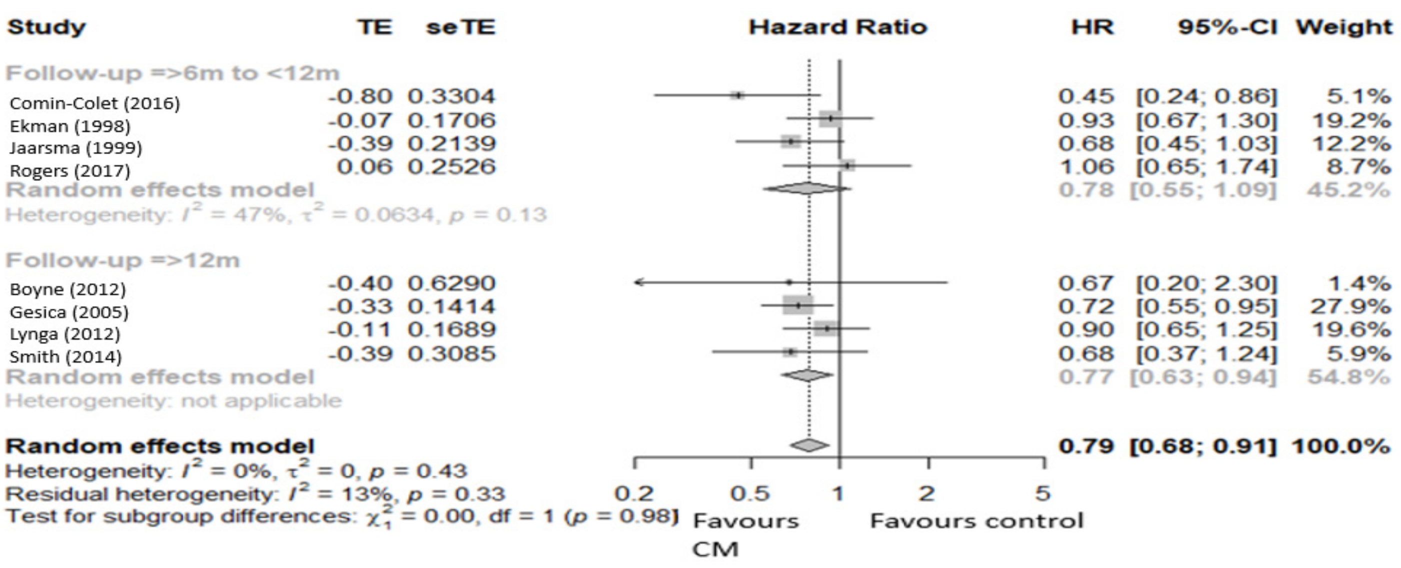

</td>
</tr>
</table>

Type of nurse-led case management delivered subgroup analysis

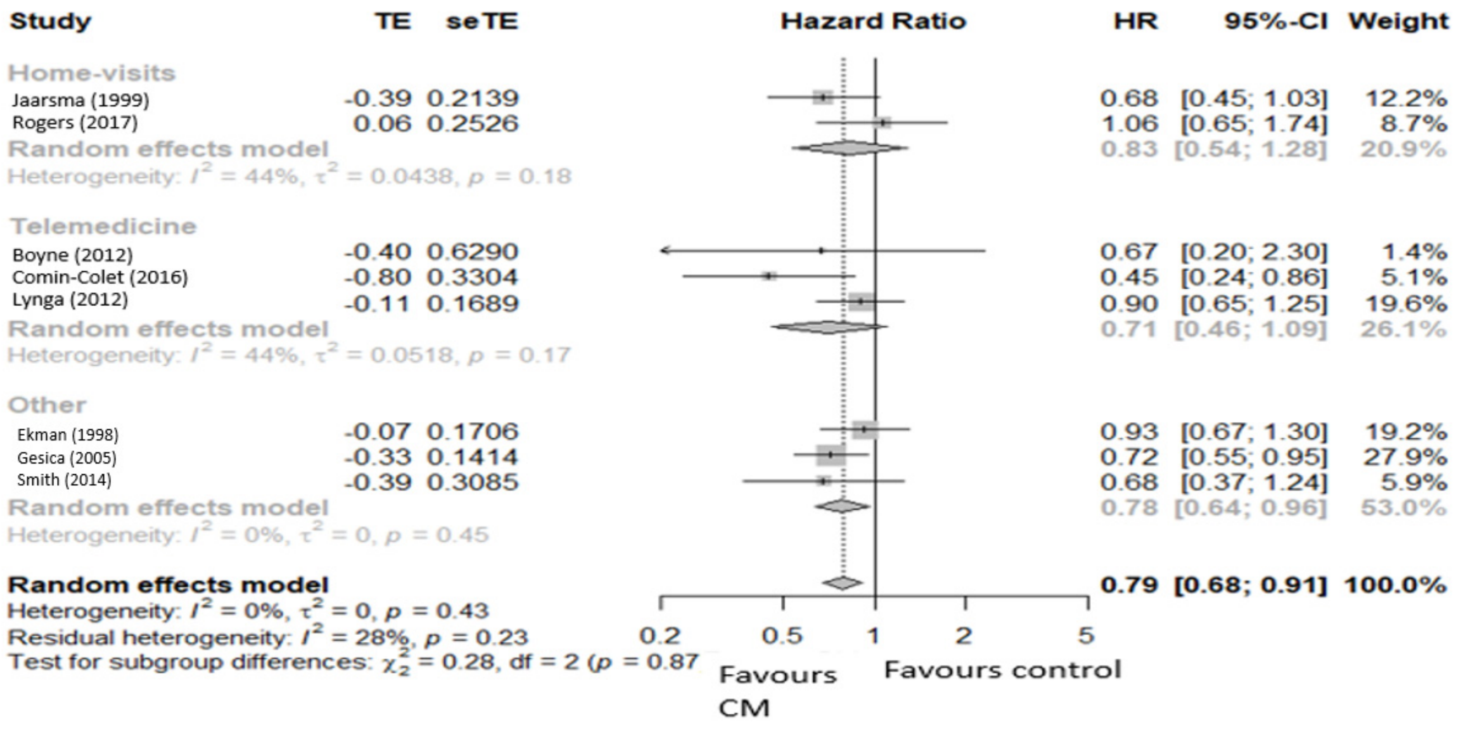

Age subgroup analysis

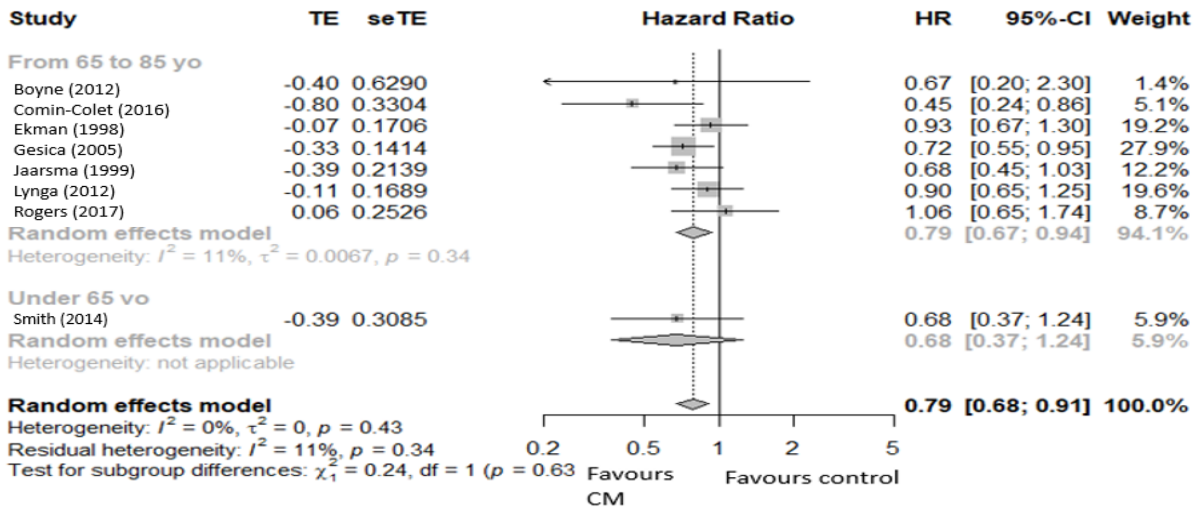

All-cause hospitalizations	Follow-up subgroup analysis<br>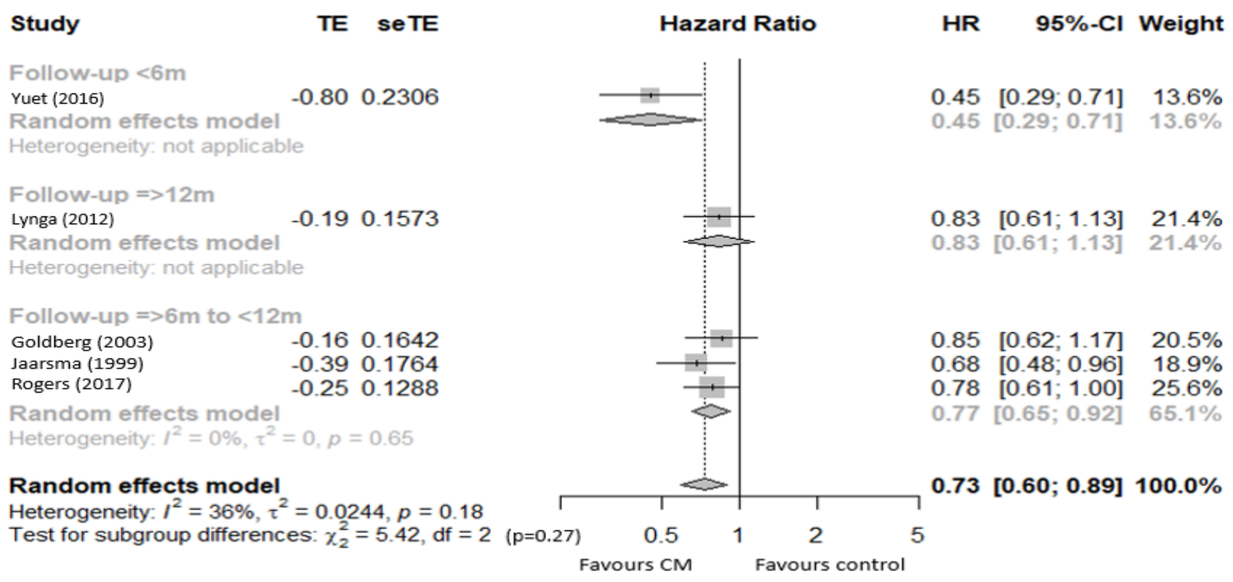

Type of nurse-led case management delivered subgroup analysis

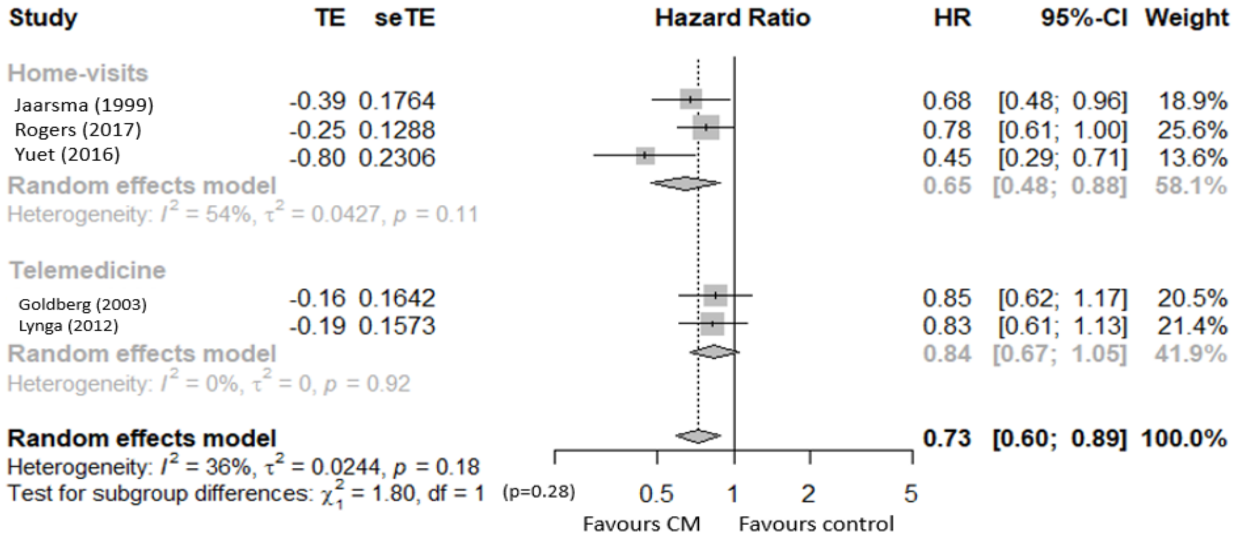

Age subgroup analysis

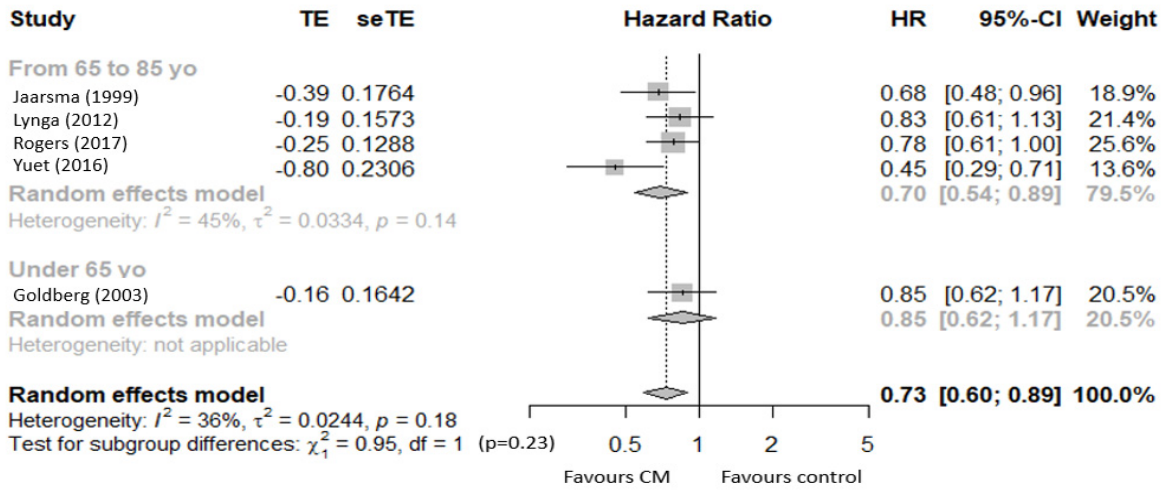

Quality of life	Follow-up subgroup analysis 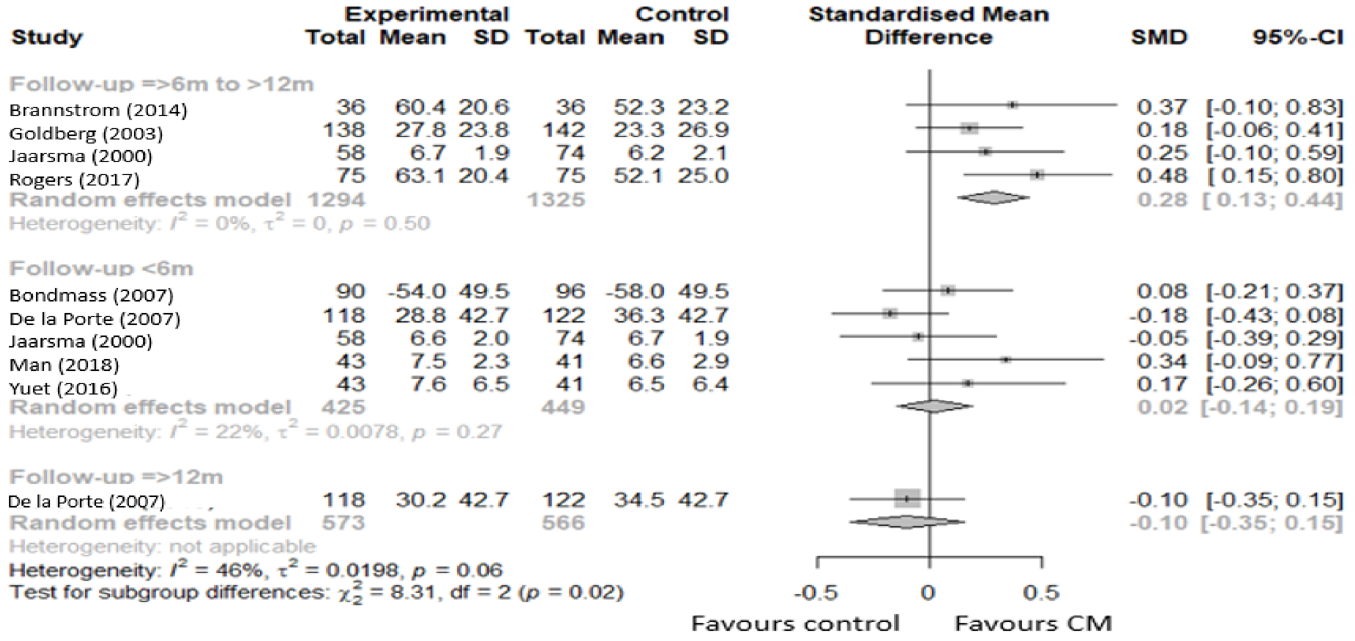

Type of nurse-led case management delivered subgroup analysis

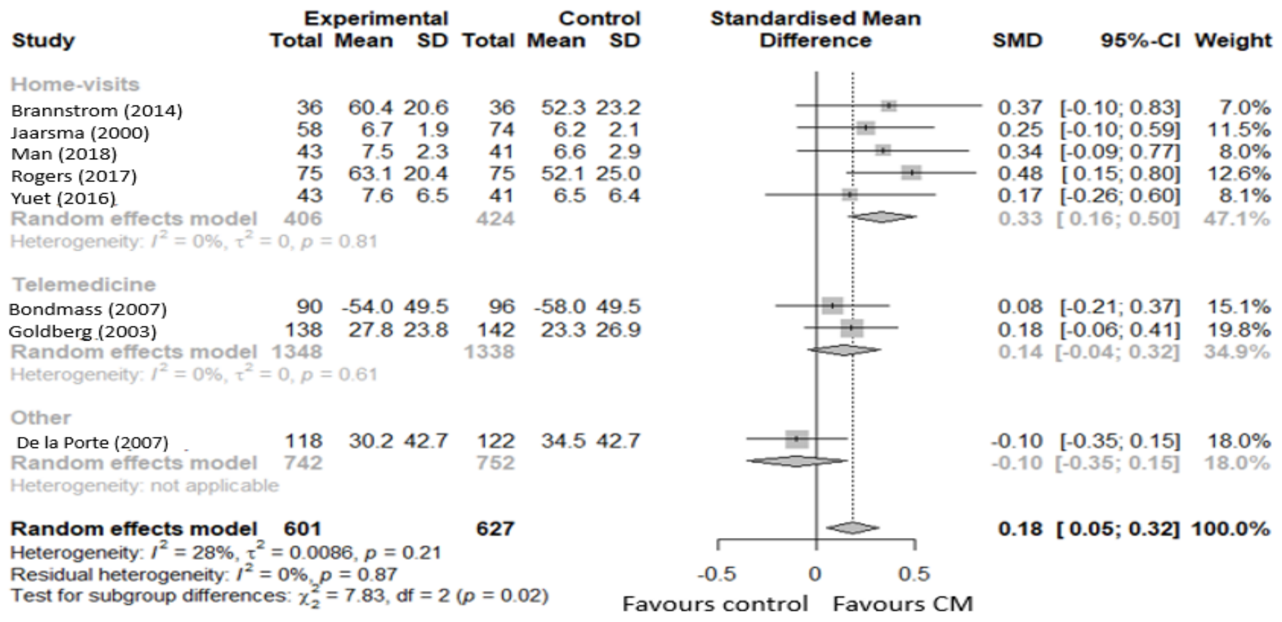

	Age subgroup analysis

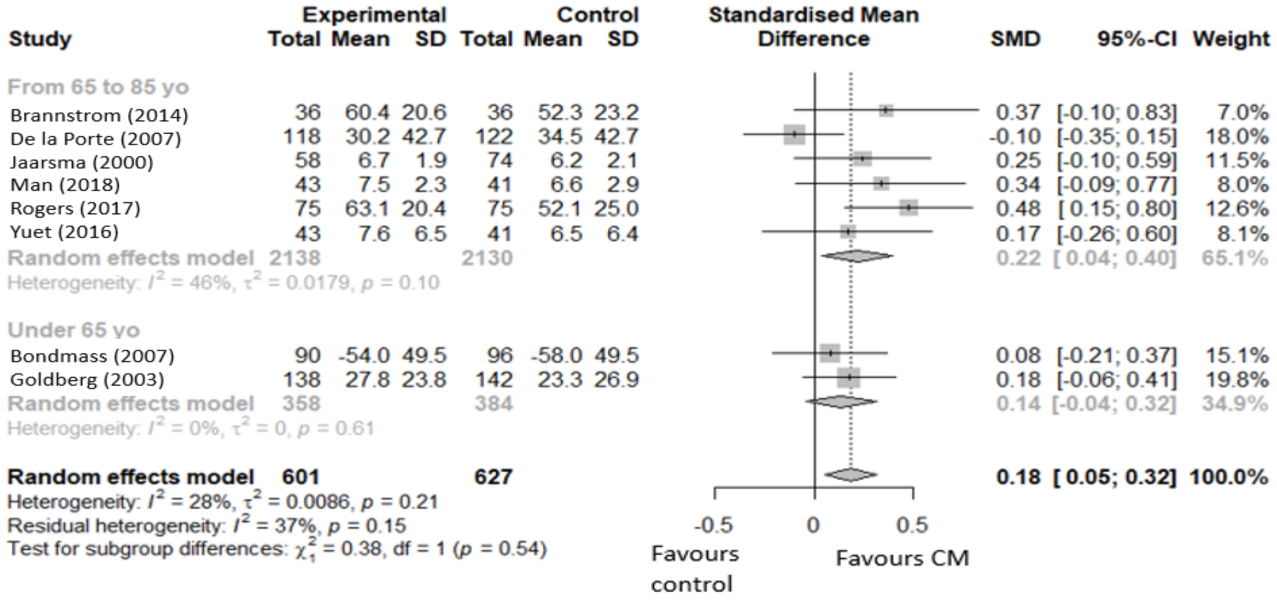

Self-care	Follow-up subgroup analysis

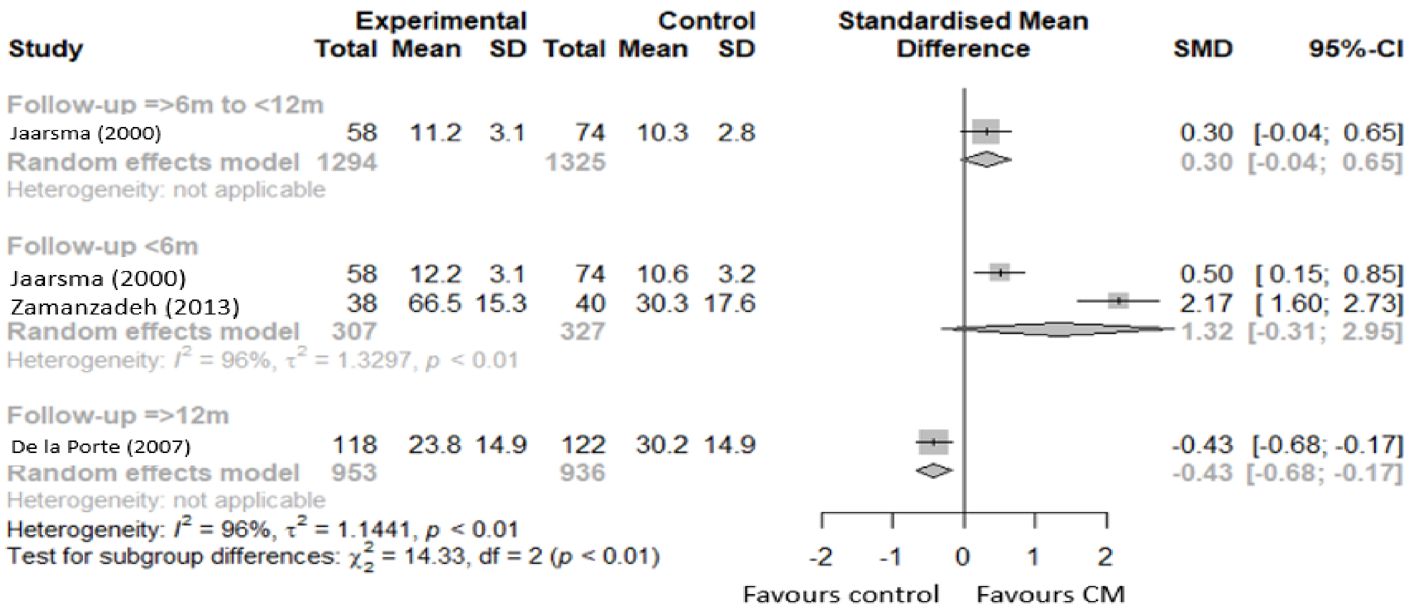

Type of nurse-led case management delivered subgroup analysis

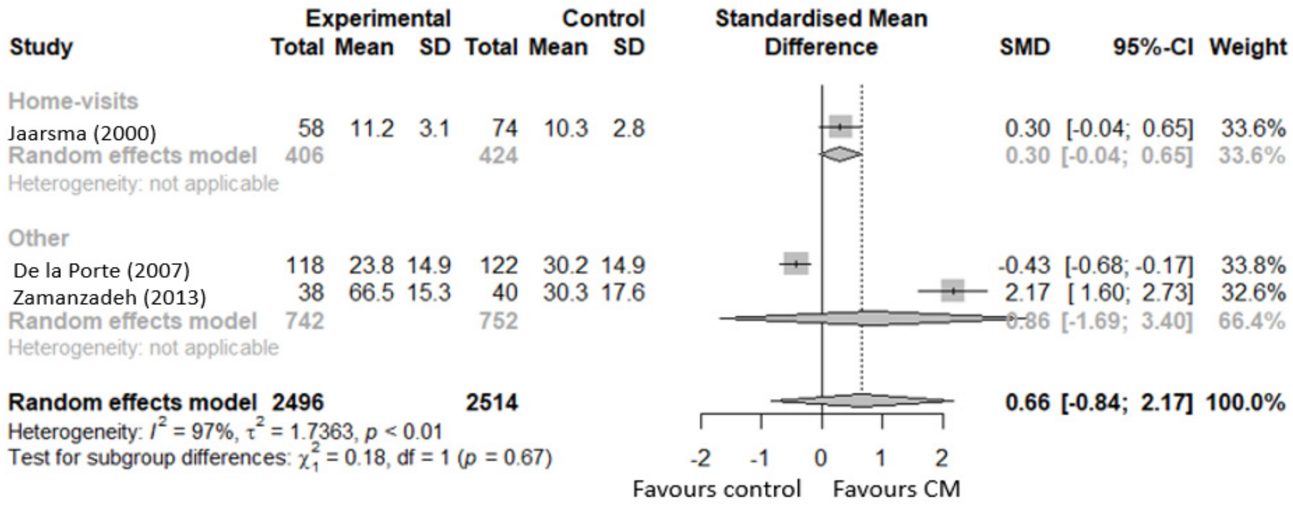

Age subgroup analysis

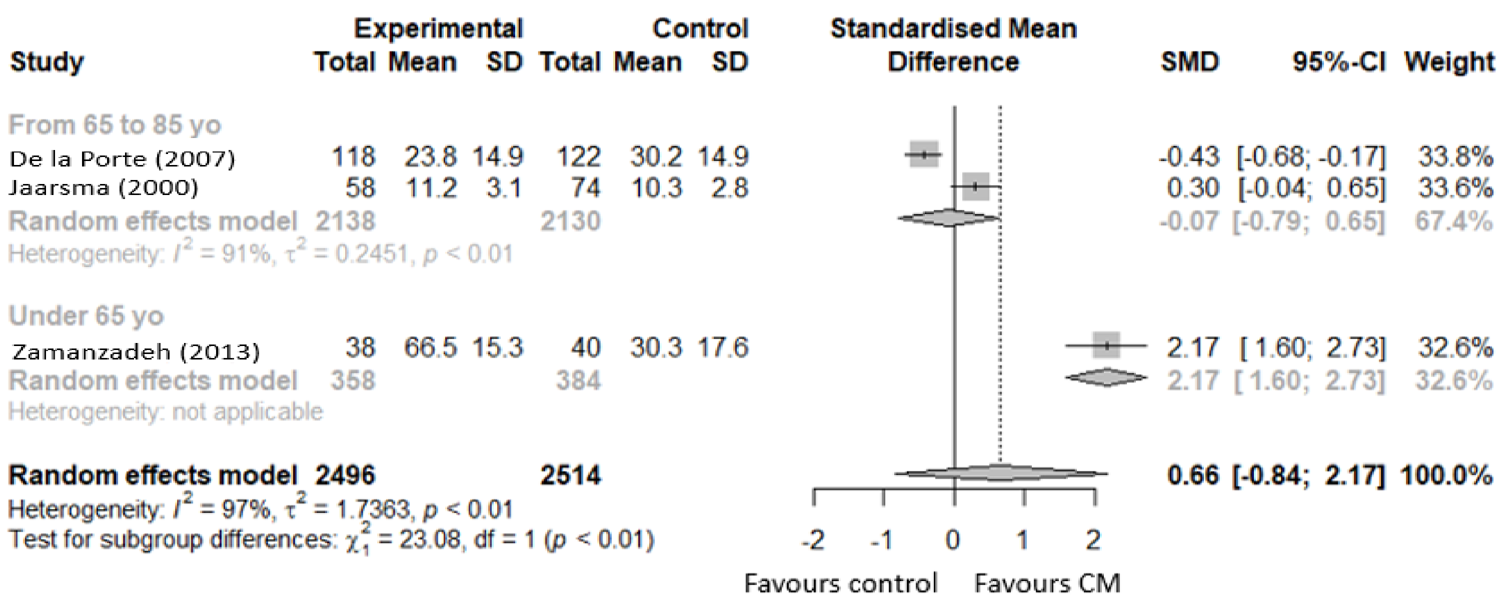

Table S5. Descriptive tables of studies with incomplete outcome data.

Hospitalizations for heart failure					
**Author, year**	**Case management (CM)**	**Number of subjects in CM group**	**Control group**	**Number of subjects in control group**	**Type of outcome reported**
De la Porte, 2015	23	118	47	122	Hospitalisations for HF and or death
Delaney, 2010	2	12	3	12	Number of events
Golberg, 2003	0.08	138	0.11	142	Average utilization
McDonald, 2001	0	35	0	35	Number of events
Fonarow, 1997	34	179	344	179	Number of events

All-cause hospitalizations					
**Author, year**	**Case management (Standard deviation)**	**Number of subjects in CM group**	**Control group**	**Number of subjects in control group**	**Type of outcome reported**
Brannstrom, 2014	15	36	53	36	Number of events
Holst, 2001	0.08 (0.28)	NI	1.05 (0.98)	NI	Average utilization
Vavouranakis, 2003	2.14 (NI)	33	1.25	28	Average utilization
Ekman, 1998	1.1 (1.3)	79	1.2 (1.5)	79	Average utilization
ONG, 2016	248	NI	223	NI	Number of events
Schellinger, 2011	34.9%	63	31.5%	715	Percentage of readmitted subjects
Shah, 1998	0.5 (NI)	17	1.2 (NI)	17	Average utilization

CM: Case management

Quality of life			
**Author, year**	**Questionnarie**	**Case Management (standard deviation)**	**Control group (standard deviation)**
Aiken, 2006	SF-36	0.16 (NI)	-0,17 (NI)
Delaney, 2010	MLHF	42.5 (17.9)	58.0 (17)
Holst, 2001	MLHF	32 (NI)	53 (NI)
Vavouranakis, 2003	MLHF	2.68 (0.034)	2.33 (0.032)

SF-36: Short Form-36 Health Survey **MLHF**: Minnesota living with heart failure
